# Targeting WEE1 in *ARID1A/TP53* Concurrent Mutant Colorectal Cancer by Exploiting R‐Loop Accumulation and DNA Repair Deficiencies

**DOI:** 10.1002/advs.202512074

**Published:** 2025-11-30

**Authors:** Chi Zhang, Yanjing Zhu, Luoyan Ai, Jingyuan Wang, Shan Yu, Yichen Wang, Xiaojing Xu, Mengling Liu, Yiyi Yu, Mengxuan Zhu, Yutong Liu, Zhenghang Xu, Haojie Zhou, Huishan Li, Qihong Huang, Qing Liu, Ke Peng, Tianshu Liu

**Affiliations:** ^1^ Department of Medical Oncology Zhongshan Hospital Fudan University Shanghai 200032 China; ^2^ Cancer Center Shanghai Key Laboratory of Oncology Target Discovery and Antibody Drug Development Zhongshan Hospital Fudan University Shanghai 200032 China; ^3^ Department of Pathology Zhongshan Hospital Fudan University Shanghai 200032 China; ^4^ Department of Oncology Zhongshan Hospital (Xiamen) Fudan University Xiamen 361000 China; ^5^ Clinical Research Center for Precision Medicine of Abdominal Tumor of Fujian Province Xiamen 361000 China; ^6^ Center of Evidence‐based Medicine Fudan University Shanghai 200032 China

**Keywords:** colorectal cancer, DNA damage repair, R‐loop, WEE1 inhibitors

## Abstract

*ARID1A*, a component of the SWI/SNF tumor suppressor complex, is frequently mutated in colorectal cancers (CRC). Here, it is found that CRC with *ARID1A/TP53* concurrent mutations is highly sensitive to WEE1 inhibitors. *ARID1A* deficiency promoted the accumulation of R‐loops, leading to replication stress. This stress, combined with the loss of G1/S and G2/M checkpoint controls due to P53 and WEE1 dysfunction, resulted in substantial DNA damage. Through chromatin accessibility sequencing, it is further revealed that *ARID1A* loss impaired *ATF3* transcription, thereby exacerbating WEE1‐inhibitor‐induced DNA damage and cell death. This preclinical evidence is supported by a phase 1b/2 trial of WEE1‐inhibitor‐based therapy in metastatic CRC patients (NCT06363552), where one patient harboring *ARID1A/TP53* concurrent mutations achieved liver lesion regression. Moreover, though CRISPR knockout screening, it is found that concurrent AKT blockade significantly augmented the antitumor effects of the WEE1 inhibitor. In conclusion, WEE1 inhibition offers a promising therapeutic strategy for *ARID1A/TP53* concurrent mutant CRC.

## Graphical abstract

*ARID1A*, a SWI/SNF complex component, is frequently mutated in colorectal cancer (CRC). CRC with *ARID1A/TP53* concurrent mutations shows marked sensitivity to WEE1 inhibition. *ARID1A* loss induces R‐loop‐mediated replication stress, impairs *ATF3* transcription, and amplifies WEE1i‐induced DNA damage, suggesting a promising therapeutic vulnerability.

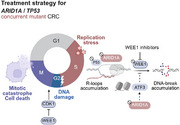

## 1 Introduction

In recent years, precision therapy for metastatic colorectal cancer (mCRC) has made remarkable strides, significantly improving survival outcomes compared to standard chemotherapy.^[^ ^1^, ^2^, ^3^ ^]^ However, treatment options remain limited for late‐line therapy.^[^ ^4^, ^5^ ^]^ The advent of next‐generation sequencing has revolutionized precision oncology, enabling the discovery of multiple targeted therapies based on a comprehensive understanding of genetic alterations.^[^ ^6^, ^7^ ^]^ While most targeted therapies focus on oncogenes, tumor suppressor genes present a persistent therapeutic challenge. This underscores the urgent need to develop effective strategies targeting tumor suppressor mutations, particularly for late‐line mCRC patients.

SWI/SNF chromatin remodeling complexes function as tumor suppressors by hydrolyzing ATP to mobilize nucleosomes, thereby regulating DNA accessibility and gene expression. SWI/SNF complexes consist of both shared ATPase subunits and subfamily‐specific subunits,^[^ ^8^, ^9^ ^]^ where *ARID1A* is the most frequently mutated subunit among different cancers, occurring in 9–11% of CRC.^[^ ^10^, ^11^ ^]^ *ARID1A* mutations are considered loss‐of‐function (LoF) events,^[^ ^12^, ^13^, ^14^ ^]^ and studies in mice have shown that *ARID1A* deficiency leads to the silencing of transcription factors essential for lineage differentiation and gives rise to colon cancer^[^ ^15^ ^]^ Typically, *ARID1A*‐mutant tumors exhibit genomic instability, marked by a high tumor mutation burden (TMB).^[^ ^14^, ^16^ ^]^ This instability is closely related to the dual impact of *ARID1A* loss in amplifying replication stress as a source of DNA damage and concurrently suppressing DNA damage repair (DDR) pathways.^[^ ^16^, ^17^, ^18^, ^19^ ^]^ Previous studies have shown that *ARID1A* deficiency were synthetic lethal with PARP inhibition and ATR inhibition,^[^ ^17^, ^20^, ^21^ ^]^ both were key regulators of DDR and replication stress response. However, clinical evidence supporting the efficacy of these treatments in CRC remains limited.

Effective DDR relies on the proper coordination of cell cycle checkpoints, which temporarily halt cell cycle progression to permit repair of damaged DNA. Among these, WEE1 acts as a key G2/M checkpoint kinase that phosphorylates CDK1 to prevent premature mitotic entry. WEE1 inhibition forces cells in the S phase to bypass the G2/M checkpoint, resulting in the accumulation of unresolved DNA damage and triggering eventual mitotic catastrophe.^[^ ^22^, ^23^, ^24^, ^25^ ^]^ WEE1 inhibitors are currently being evaluated in phase II clinical trials, showing the best efficacy in ovarian and endometrial cancers.^[^ ^26^, ^27^, ^28^ ^]^ However, efficacy is quite limited in other solid tumors, including CRC.^[^ ^29^ ^]^ While *TP53* mutations were initially proposed as a predictive biomarker for WEE1 inhibitors, clinical data have yielded inconsistent results regarding their predictive value.^[^ ^25^ ^]^ Therefore, this underscores the need to identify more precise therapeutic biomarkers as well as potent combination strategies to enhance the efficacy of WEE1 inhibitors.

Here, we demonstrate that WEE1 inhibition is synthetic lethal in CRC with *ARID1A/TP53* concurrent mutations, where the clinical efficacy of the WEE1 inhibitor was also observed in one patient harboring these mutations. Additionally, alternative combination strategies were profiled with CRISPR screening, revealing that AKT inhibitors can further enhance the efficacy of WEE1 inhibitors.

## 2 Results

### 2.1 WEE1 Inhibitors Selectively Target *ARID1A*/*TP53* Concurrent Mutant CRC

**Figure 1:**
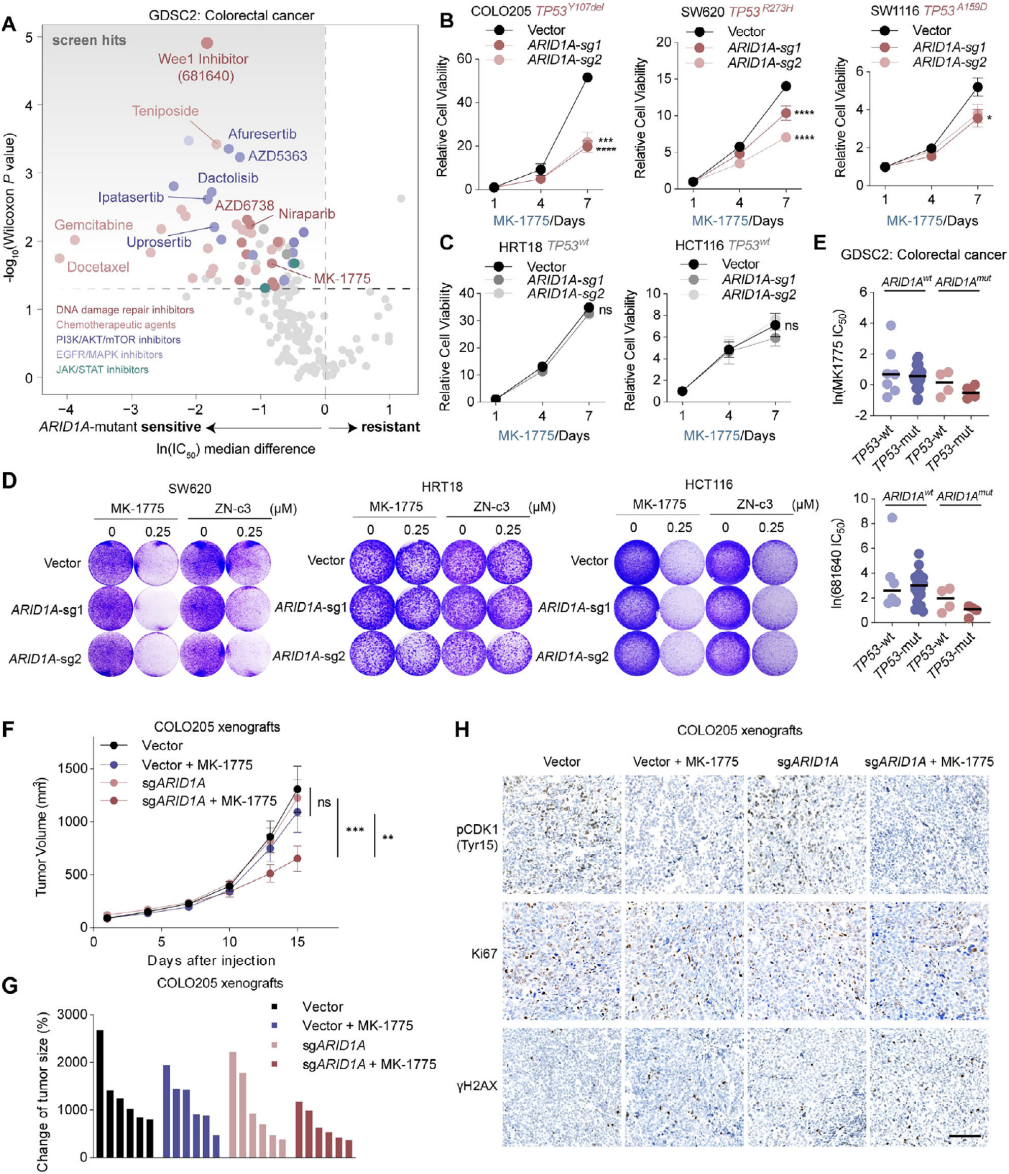
WEE1 inhibitors selectively target *ARID1A/TP53* concurrent mutant CRC. A) Median IC_50_ difference of 185 compounds in CRC cell lines from the GDSC2 project. A negative value indicates preferential sensitivity to the corresponding compound. (B‐C) Cell proliferation assays of *TP53*‐mutant (B) or *TP53* wildtype (C) cells treated with 0.25 µm MK‐1775 for the indicated days. The results are representative of three independent experiments, each done in triplicate. Data are presented as mean ± SD; ns, not significant; ^*^ *p* < 0.05, ^***^ *p* < 0.001, ^****^ *p* < 0.0001 (two‐way ANOVA). (D) Crystal violet staining of the indicated cell lines treated with MK‐1775 or ZN‐c3 for 7–10 days (n = 3). (E) GDSC2 subgroup analysis of MK‐1775 (top) or 681640 (bottom) according to *ARID1A/TP53* mutation status in CRC cells. (F) Growth curves of COLO205 xenografts in BALB/c nude mice treated with either MK‐1775 or vehicle. MK‐1775 (60 mg kg^−1^) was administered via gavage (n = 6). Data are shown as mean ± SEM; ns, not significant; ^**^ *p* < 0.01, ^***^ *p* < 0.001 (two‐way ANOVA). (G) Waterfall plot showing the tumor volume change (at day 15) relative to baseline volume (at day 1). Each bar represents one xenograft tumor. (H) Representative immunohistochemical staining of pCDK1, Ki‐67, and γH2AX staining of the COLO205 xenograft tumors. Scale bar = 100 µm.

To uncover drugs that specifically target *ARID1A*‐mutant CRC, we mined data from the *Genomics of Drug Sensitivity in Cancer* (GDSC2) project.^[^ ^30^ ^]^ The results revealed that *ARID1A*‐mutant CRC cells were more sensitive to DNA‐damage‐inducing drugs, including chemotherapeutic agents and DDR modulators^[^ ^17^, ^21^ ^]^ (**Figure** **1**A). Among them, the WEE1 inhibitor displayed the most significant IC_50_ difference between *ARID1A*‐mutant and wild‐type CRC cell lines [ln (IC_50_) median difference = −1.85, *P* < 0.0001] (Figure 1A, Table S1, Supporting Information). Notably, other types of cancers with frequent *ARID1A* mutations demonstrated much lower sensitivity to WEE1 inhibitor and other drugs (Figure S1, Supporting Information), highlighting the unique therapeutic potential of targeting *ARID1A* mutations in CRC.

As *ARID1A* mutation is typically considered as LoF, we established *ARID1A* knockout (*ARID1A^KO^*) cell lines to mimic the effect of *ARID1A* mutation (Figure S2B, Supporting Information), and assessed relative cell viability after WEE1 inhibitors (MK‐1775 and ZN‐c3) treatment. *ARID1A^KO^* had a minimal effect on cell proliferation (Figure S2C, Supporting Information). Notably, *ARID1A^KO^* CRC cells responded differently to WEE1 inhibition. After WEE1 inhibitor treatment, a significant growth inhibition occurred in *ARID1A^KO^* COLO205, SW620, and SW1116 cells compared to their vector cells, whereas such a trend was absent in *ARID1A^KO^* HRT18 and HCT116 cells (Figure 1B–D; Figure S2D, Supporting Information). We then compared the genetic backgrounds of these cell lines and found that *ARID1A^KO^* sensitized WEE1 inhibitors only in *TP53*‐mutant cells, but not in *TP53* wild‐type ones (Figure S2A, Supporting Information). Subgroup analysis from the GDSC2 also confirmed that *ARID1A*‐mutant CRC cells with concurrent *TP53* mutations generally exhibited a lower IC_50_ of WEE1 inhibitors compared to *TP53* wildtype cells (Figure 1E).

Next, we evaluated the efficacy of WEE1 inhibitor MK‐1775 in *TP53‐*mutant COLO205 xenografts with or without *ARID1A^KO^*. MK‐1775 was well‐tolerated according to the weight of the mice in both groups (Figure S3A, Supporting Information). In line with in vitro experiments, *ARID1A^KO^* tumors had a better response to MK‐1775 compared to *ARID1A* wildtype group (Figure 1F–G; Figure S3B, Supporting Information). To assess the on‐target effect, we performed phospho‐CDK1 (pCDK1), γH2AX, and Ki‐67 immunohistochemical staining. Treatment with MK‐1775 led to a significant reduction in pCDK1 at Tyr15, and a moderate increase in DNA damage marker γH2AX (Figure 1H; Figure S3C, Supporting Information). Together, these data suggested that WEE1 blockade potently inhibited *ARID1A/TP53* concurrent mutant CRC.

### 2.2 WEE1 Inhibition Induces Micronuclei Formation and Pan‐Nuclear γH2AX Accumulation in *ARID1A/TP53* Concurrent Deficient CRC Cells

**Figure 2:**
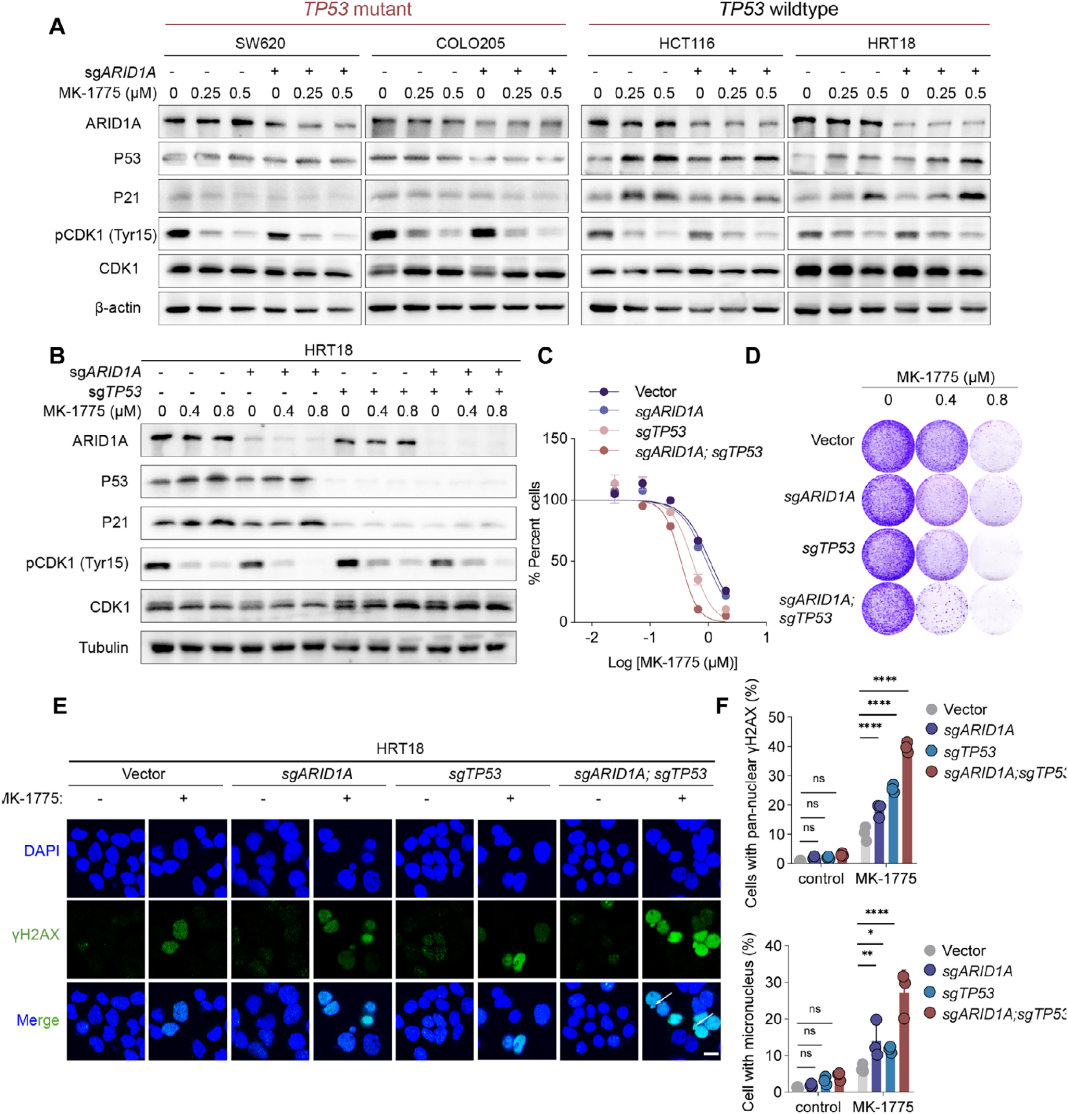
WEE1 inhibition induces micronuclei formation and pan‐nuclear γH2AX accumulation in *ARID1A/TP53* concurrently deficient CRC cells. A) Immunoblot analysis of *ARID1A* knockout (sg1) CRC cell lines treated with WEE1 inhibitors. COLO205 (*TP53*‐mutant), SW620 (*TP53*‐mutant), HCT116 (*TP53* wildtype), and HRT18 (*TP53* wildtype) cells were treated with MK‐1775 at the indicated concentrations for 48 h (n = 2). B) Immunoblot analysis of *TP53* (sg3) and/or *ARID1A* (sg1) knockout cells. HRT18 cells were treated with MK‐1775 at the indicated concentrations for 48 h (n = 2). C) Dose‐response curves of HRT18 treated with MK‐1775 for 5 days. Results are representative of three independent experiments performed in triplicate. Data are presented as mean ± SD. D) Crystal violet staining of HRT18 cells, treated with MK‐1775 at the indicated concentration for 7–10 days (n = 3). E) Representative immunofluorescent images of HRT18 cells after 0.5 µm MK‐1775 treatment for 24 h. White arrows indicate micronucleated cells. Scale bar = 15 µm. F) Proportion of pan‐nuclear γH2AX cells (top) and micronucleated cells (bottom) in E (n=3). Data are shown as mean ± SD; ns, not significant; *p* < 0.05, ^*^ *p* < 0.05, ^**^ *p* < 0.01, ^****^ *p* < 0.0001 (one‐way ANOVA).

To investigate the cellular response and DNA damage induced by WEE1 inhibition, we examined the activity of G2/M cell cycle checkpoints and DNA break signals. We found that WEE1 inhibition by MK‐1775 or ZN‐c3 suppressed CDK1 phosphorylation across different genetic backgrounds (**Figure** **2**A; Figure S4A,B, Supporting Information). However, we found a robust activation of P53‐P21 pathway in *TP53* wildtype cells, but not in the *TP53*‐mutant SW620 and COLO205 cells (Figure 2A; Figure S4A,B,Supporting Information). It has been reported that P53 activation resulted in G1/S cell cycle arrest, thereby preventing further DNA damage caused by WEE1 inhibition.^[^ ^31^, ^32^, ^33^ ^]^ This process also involves the transactivation of P21, which helps reduce DNA damage during S‐phase and contributes to resistance of WEE1 inhibition.^[^ ^34^ ^]^

We then generated *TP53* knockout (*TP53^KO^*) cells in the *ARID1A^KO^* background, given that *TP53* mutations predominantly result in LoF (Figure 2B; Figure S4C, Supporting Information). Consistently, we found that *TP53^KO^* cells failed to activate P21 (Figure 2B; Figure S4C, Supporting Information), leading to an impaired viability under WEE1 inhibitor treatment, compared with *TP53* wildtype cells (Figure 2C,D; Figure S4D,E, Supporting Information). Besides, *ARID1A/TP53* double knockout further inhibited cell growth under WEE1 inhibitor treatment, compared to *ARID1A* or *TP53* knockout alone (Figure 2C,D; Figure S4D,E, Supporting Information).

We asked whether such effects in double knockout cells were attributed to enhanced DNA damage and mitotic catastrophe. Compared to *TP53* or *ARID1A* knockout alone, *ARID1A/TP53* double knockout cells exhibited significantly stronger signal of γH2AX induced by WEE1 inhibition (Figure 2E,F). Following the observed DNA damage, cells undergoing mitotic catastrophe exhibited markedly abnormal nuclear morphology, characterized by extensive micronuclei formation (Figure 2E,F). In *ARID1A/TP53* concurrent deficient cells (HRT18 double knockout cell and COLO205 *ARID1A^KO^* cell), WEE1 inhibitor treatment led to frequent formation of micronuclei (Figure 2E,F; Figure S4F, Supporting Information).

These findings highlight that the concurrent disruption of *ARID1A* and *TP53* compromises genome integrity and mitotic fidelity upon WEE1 inhibition, driving synthetic lethality through enhanced DNA damage and mitotic failure.

### 2.3 R‐Loop Accumulation and Replication Stress Induced by *ARID1A* Knockout Sensitized CRC Cells to WEE1 Inhibition

Previous studies have reported that *ARID1A* deficiency can increase CDK1 activity either by elevation of total CDK1,^[^ ^35^ ^]^ or by dephosphorylation of CDK1 through CDC25C.^[^ ^36^ ^]^ We found *ARID1A^KO^* had minimal effect on total or phosphor‐CDK1 in our CRC models (Figure 2A,B; Figure S4A–C, Supporting Information).

**Figure 3:**
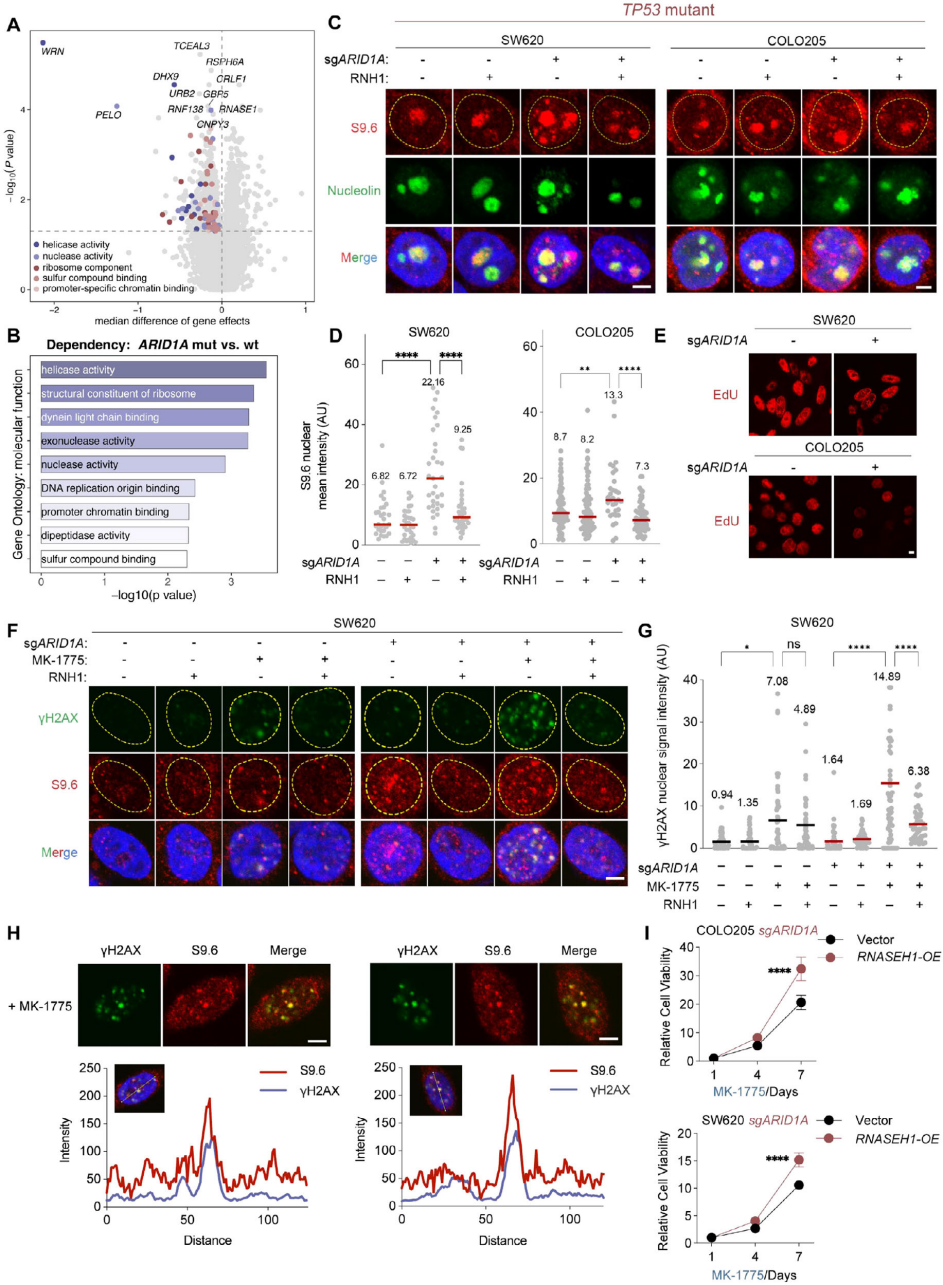
R‐loop accumulation and replication stress induced by *ARID1A* knockout sensitized CRC cells to WEE1 inhibition. A) Median difference of gene dependencies across CRC cell lines from the DepMap project. A negative value indicates that knockout of a certain gene impairs cell viability. (B) Gene Ontology (GO) analysis of *ARID1A*‐mutant dependent genes, ranked by *P* values. (C) Representative immunofluorescence images showing nuclear S9.6 signal intensity in SW620 (left) and COLO205 (right) (n = 3). Scale bar, 5 µm. RNH1, short for RNASEH1 (overexpression). (D) Quantification of nuclear S9.6 signal intensity in (C), SW620 (left) and COLO205 (right). Considering that S9.6 has a colocalization signal with nucleolin, S9.6 mean intensity was calculated after subtracting the nucleolin signal.^[^ ^19^, ^49^ ^]^ Data are presented as scatter plots (n = 3). Median values are indicated by red lines. ^**^ *p* < 0.01, ^****^ *p* < 0.0001(two‐tailed Wilcoxon rank‐sum test). AU, arbitrary units. (E) Representative EdU staining images in SW620 and COLO205 cells. Scale bar, 5 µm. (F) Representative immunofluorescence images showing nuclear S9.6 and γH2AX signal intensity in SW620 cells treated with or without 0.5 µm MK‐1775. Scale bar, 5 µm (n = 3). (G) Quantification of nuclear S9.6 and γH2AX signal intensity in (F). Data are presented as scatter plots (n = 3). Median values are indicated by red lines. ns, not significant; ^**^ *p* < 0.01, ^****^ *p* < 0.0001 (two‐tailed Wilcoxon rank‐sum test). (H) Representative colocalization of nuclear S9.6 and γH2AX signals in SW620 cells treated with 0.5 µm MK‐1775. Scale bar, 5 µm. (I) Relative cell viability of COLO205 and SW620 cells overexpressing RNASEH1 and treated with 0.5 µm MK‐1775 for the indicated days. Results are representative of three independent experiments performed in triplicate. Data are displayed as mean ± SD; ^****^ *p* < 0.0001 (two‐way ANOVA).

To understand the molecular mechanism of *ARID1A^KO^* cells responding to WEE1 inhibition, we mined DepMap data^[^ ^37^ ^]^ to screen dependent genes in *ARID1A*‐mutant CRC cells (**Figure** **3**A; Table S2, Supporting Information). Gene Ontology (GO) enrichment analysis of these dependent genes revealed that *ARID1A*‐mutant cells were more reliant on DNA‐RNA helicases (Figure 3B), which were recently recognized to dissolve DNA‐RNA hybrids (R‐loops). R‐loops are formed when nascent RNA hybridizes with DNA, displacing the non‐template strand as single‐stranded DNA (ssDNA), which usually occurs from transcription‐replication conflict.^[^ ^38^, ^39^, ^40^ ^]^ Helicases such as *WRN* and *DHX9* were among the top hits in *ARID1A*‐mutant CRC cells (Figure 3A), which were known to prevent the accumulation of R‐loops, providing therapeutic potential in cancer.^[^ ^41^, ^42^, ^43^ ^]^ Based on these findings, we hypothesized that *ARID1A*‐mutant cells might produce excessive R‐loops, which need to be dissolved by these helicases to prevent potential DNA damage induced by WEE1 inhibition.

To confirm these findings in our model, we first detected R‐loop accumulation in *ARID1A^KO^ TP53‐*mutant cells through S9.6 immunofluorescence. As expected, R‐loops were significantly more abundant in COLO205 and SW620 cells after *ARID1A^KO^* (Figure 3C,D). This accumulation was reversed upon overexpressing of *RNASENH1* (RNH1), a R‐loop resolution helicase^[^ ^19^ ^]^ (Figure 3C,D; Figure S5A, Supporting Information). Next, we evaluated replication stress in *ARID1A^KO^* cells by EdU staining and flow cytometry.^[^ ^19^ ^]^ It showed that EdU staining was significantly weaker in *ARID1A^KO^* SW620 and COLO205 cells (Figure 3E; Figure S5B, Supporting Information), and cell cycle analysis revealed a clear prolongation of the S phase in *ARID1A^KO^* cells (Figure S5C, Supporting Information), indicating heightened replication stress following *ARID1A^KO^*.

Cancer cells with excessive replication stress are highly dependent on cell cycle checkpoints to resolve replication stress and prevent DNA damage.^[^ ^33^, ^44^, ^45^ ^]^ We hypothesized that WEE1 inhibition forces *ARID1A^KO^* cells to enter mitosis without restoring stalled replication forks, causing irreversible DNA damage. Compared to wildtype cells, *ARID1A^KO^* cells displayed stronger γH2AX staining following the treatment of WEE1 inhibitor (Figure 3F,G). We next investigated whether the increased DNA damage in WEE1‐inhibitor‐treated *ARID1A^KO^* cells was R‐loop dependent. In *ARID1A^KO^* cells, the γH2AX intensity was significantly reduced after overexpressing *RNASEH1* (Figure 3F,G). The colocalization plot also indicated that γH2AX signal tended to culminate in S9.6 positive loci (Figure 3H). Moreover, cell viability assays indicated that overexpression of *RNASEH1* reduces the sensitivity of *ARID1A^KO^* cells to WEE1 inhibitors, while the sensitivity of *ARID1A* wild‐type cells remains unaffected (Figure 3I). These results confirmed that WEE1 inhibition exacerbates R‐loop‐induced replication stress, leading to substantial DNA damage in *ARID1A^KO^* cells.

To capture the response of WEE1 inhibition in *ARID1A^KO^* cells, we performed RNA sequencing in *ARID1A^KO^* and vector cells of COLO205. Differentially expressed genes (DEGs) were analyzed to compare differences between WEE1 inhibitor treatment and DMSO (Supplementary Table S3, Supporting Information). Interestingly, the GO enrichment of DEGs revealed that WEE1 inhibition upregulated helicases involved in resolving DNA‐RNA hybrids^[^ ^46^, ^47^, ^48^ ^]^ only in *ARID1A^KO^* cells, but not in *ARID1A* wild‐type cells (Figure S5D,E, Supporting Information). We postulated that upregulation of helicases represents an adaptive process for *ARID1A^KO^* cells in response to substantial DNA damage induced by R‐loop accumulation. Together, these findings suggested that *ARID1A^KO^* induces R‐loop accumulation, which primes substantial DNA damage for WEE1 inhibition.

### 2.4 *ARID1A* Knockout Disrupts *ATF3* Transcription and Impairs Regulation of P53 and UV Response Pathways

As a key member of the chromatin remodeling complex family, *ARID1A* can also influence the expression of various downstream genes by modulating chromatin accessibility. To comprehensively investigate the mechanisms underlying the response of *ARID1A/TP53* knockout cells to WEE1 inhibition, we performed ATAC‐seq and RNA‐seq on *ARID1A/TP53* double knockout and *TP53* knockout HRT18 cells.

**Figure 4:**
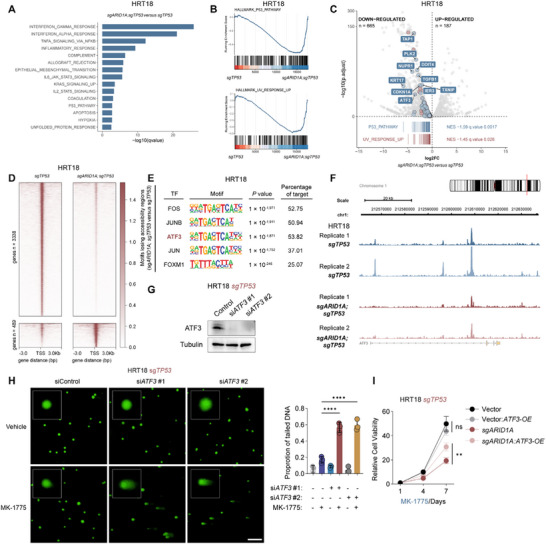
*ARID1A* knockout disrupts *ATF3* transcription and impairs the regulation of P53 and UV response pathways. A) Summary of hallmark gene GSEA analysis of RNA‐seq comparing *ARID1A/TP53* double knockout cells with *TP53* knockout cells, ranked by *P* values. (B) GESA plots showing normalized enrichment score (NES) of P53 and UV response gene sets. (C) Volcano plot displaying log_2_(fold change) and ‐log_10_(p value) of differentially expressed genes in *ARID1A/TP53* double knockout cells vs *TP53* knockout cells. (D) Heatmap showing lost and gained chromatin accessibility peaks identified by ATAC‐seq in *ARID1A/TP53* double knockout cells vs *TP53* knockout cells. (E) HOMER motif enrichment analysis of regions with decreased chromatin accessibility in *ARID1A/TP53* double knockout cells. (F) Relative peak signal of *ATF3* locus in HRT18 cells. (G) Immunoblot analysis of *ATF3* expression in HRT18 sg*TP53* cells, following si‐*ATF3* transfection. (H) Representative immunofluorescence images of the alkaline comet assay performed in the indicated HRT18 cells with two ATF3 siRNAs (treatment: DMSO/0.5 µm MK‐1775, 24 h). Scale bar, 50 µm. The right panel calculates the proportion of COMET‐tailed cells (n=3). Data are presented as mean ± SD; ^****^ *p* < 0.0001 (one‐way ANOVA). (I) Cell proliferation assays of HRT18 sg*TP53* cells treated with 0.5 µm MK‐1775 for the indicated days. The results are representative of three independent experiments, each done in triplicate. Data are presented as mean ± SD; ns, not significant; ^**^ *p* < 0.01 (two‐way ANOVA).

We first performed pathway enrichment analysis on the differentially expressed genes from the RNA‐seq data. Interestingly, we found that even in the context of *TP53^KO^*, *ARID1A^KO^* further downregulated the P53 signaling pathway (**Figure** **4**A–C). In addition, we observed a marked downregulation of pathways involved in the immediate response to DNA damage, such as the UV response pathway (Figure 4B,C). Though *ARID1A* primarily functions as a signaling protein involved in HR to help DNA damage repair, our findings suggest that *ARID1A* may also influence the DDR pathway through transcriptional regulation.

Next, we analyzed the ATAC‐seq data to identify the altered activity of key transcription factors (TF) under *ARID1A^KO^*. We first observed that *ARID1A^KO^* caused a widespread decrease in chromatin accessibility, with 3338 genes significantly downregulated and only 489 genes upregulated (Figure 4D). Annotation of differential peaks revealed that *ARID1A* primarily associates with distal regulatory regions of genes with reduced chromatin accessibility (Figure S6A, Supporting Information), which was in concordance with a previously reported role in modulating enhancer activity.^[^ ^15^ ^]^ Using HOMER2 for transcription factor motif analysis of the differential peaks, we found that *ARID1A^KO^* notably suppressed the binding motifs of several TFs, including *FOS*, *JUN*, *JUNB*, *ATF3*, and *FOXM1* (Figure 4E). The ATAC‐seq and RNA‐seq results showed strong correlation, further supporting the reliability of the findings (Figure S6B,C, Supporting Information).

Interestingly, we found that *ATF3* showed both decreased transcriptional expression and reduced chromatin accessibility in the *ARID1A^KO^* group (Figure S6C, Supporting Information). The ATAC peak profiles indicated that the openness signal of *ATF3* region is markedly reduced under *ARID1A^KO^* (Figure 4F). In HRT18 cells, we also found that *ARID1A^KO^* significantly downregulated the expression of *ATF3* (Figure S6D, Supporting Information). Previous studies have reported that *ATF3* may be involved in the regulation of the P53 and UV pathways, and its downregulation may lead to increased DNA damage.^[^ ^50^, ^51^ ^]^ Consistently, the chromatin binding of key genes in the P53 and UV pathways, such as *CDKN1A* and *GADD45A*, is markedly reduced in *ARID1A^KO^* cells (Figure S6C, Supporting Information).

To evaluate the impact of *ATF3* on DNA damage, we knocked down *ATF3* with siRNA in the *TP53^KO^* and *ARID1A* wild‐type HRT18 cell to determine whether it phenocopied the effect of *ARID1A^KO^* (Figure 4G). Cells were then treated with MK‐1775, and DNA damage accumulation was directly assessed using the alkaline comet assay. The results showed that *ATF3* knockdown markedly enhanced WEE1‐inhibitor‐induced DNA damage compared with the control group, as evidenced by a higher proportion of tailed DNA in *ATF3* knockdown cells (Figure 4H). These findings suggest that *ARID1A* loss may promote DNA damage accumulation partly by impairing DNA repair through *ATF3* regulation.

We speculated that *ATF3*‐induced abnormalities in DNA damage might influence the efficacy of WEE1 inhibition. Consistent with this, overexpression of *ATF3* in *ARID1A^KO^* cells markedly reduced sensitivity to MK‐1775, resulting in enhanced cell viability compared with the control group (Figure 4I; Figure S6D,E, Supporting Information). In contrast, *ATF3* knockdown in *ARID1A* wild‐type cells significantly increased sensitivity to WEE1 inhibitor (Figure S6F, Supporting Information).

Therefore, we conclude that *ARID1A^KO^* disrupts *ATF3* transcription modulation and inhibits P53/UV pathway activity, which further impairs DNA damage repair and contributes to the enhanced sensitivity to WEE1 inhibition (Figure S6G, Supporting Information).

### 2.5 Clinical Efficacy of WEE1 Inhibition in a Colorectal Cancer Patient with *ARID1A/TP53* Concurrent Mutations

Based on the preclinical results above, we asked whether WEE1 inhibitor would also be effective in patients harboring *ARID1A/TP53* concurrent mutation. In an ongoing phase 1b/2 trial, a novel WEE1 inhibitor, SC0191, is being tested in mCRC patients who have progressed after at least 2 lines of therapy (NCT06363552) (Figure S7A, Supporting Information). Currently, 13 patients have been recruited, and tumor biopsies at baseline were collected for whole‐exome sequencing (WES) (Figure S7B, Supporting Information). Notably, patient #12 was found to have *ARID1A/TP53* concurrent mutation (Figures S7B,D, and E, Supporting Information).

**Figure 5:**
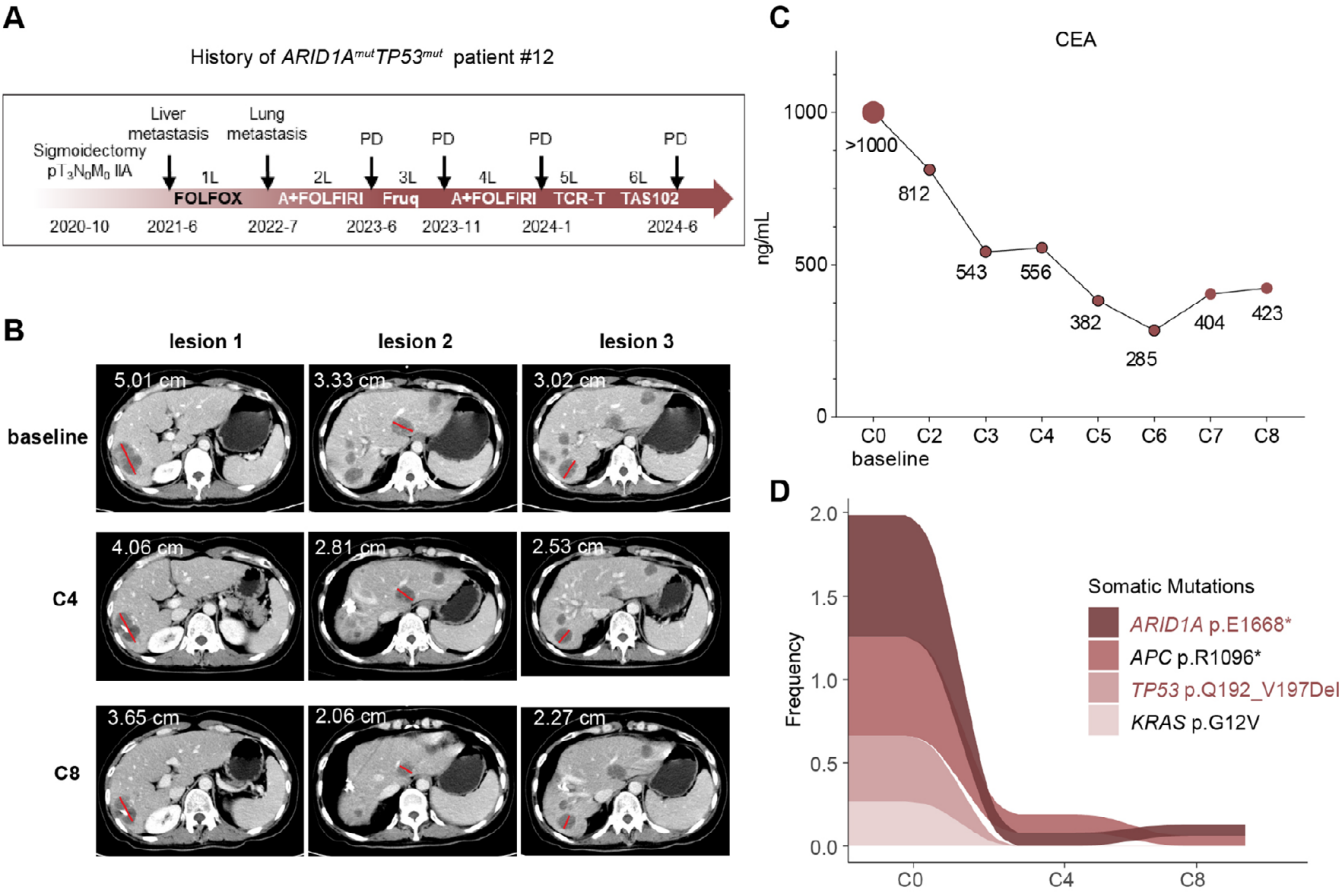
Clinical efficacy of WEE1 inhibition in a colorectal cancer patient with *ARID1A/TP53* concurrent mutations. A) Treatment timeline of patient #12 harboring *ARID1A/TP53* concurrent mutations. (B) Computed tomography (CT) images of the liver metastatic lesions at treatment cycles C0, C4, and C8 of the treatment in patient #12. (C) Serum carcinoembryonic antigen (CEA) levels at the indicated treatment cycles in patient #12. (D) Mutation evolution maps of *ARID1A, APC, TP53*, and *KRAS* in patient #12, as detected by whole‐exome sequencing (WES) of circulating tumor DNA (ctDNA) from peripheral blood. A, Bevacizumab; Fruq, Fruquintinib; PD, progressive disease.

Patient #12 was diagnosed with sigmoid colon adenocarcinoma and received sigmoidectomy in October 2020 with a tumor stage of pT_3_N_0_M_0_ IIA. Liver metastasis developed in June 2021, when the patient began to receive palliative regimens (**Figure** **5**A). After progression after 6 lines of therapy, the patient was recruited into this trial and was randomized into SC0191 plus bevacizumab group. The WES of ctDNA collected at the baseline for the trial revealed a high mutation frequency of *ARID1A* and *TP53* (Figure 5D). Notably, the Computed Tomography (CT) evaluation indicated a stable disease (SD) based on RECIST1.1, with continuous tumor regression in multiple liver metastasis lesions at C4 and C8 (Figure 5B). The level of carcinoembryonic antigen (CEA) had also been decreasing dramatically (Figure 5C). In addition, we monitored ctDNA during the treatment to evaluate molecular responses. Overall, ctDNA mutation burden decreased significantly after SC0191 treatment (Figure S7C, Supporting Information). We focused on the mutation abundance of the four most frequent driver genes (ARID1A^E1668*^, APC^R1096*^, TP53^Q192‐V197Del^, KRAS*^G12V^*) in this patient. Notably, the frequency of all four mutations decreased at C8, with ARID1A^E1668*^ dropping from 72% to 7.9% (Figure 5D).

The combination of WEE1 inhibitor and bevacizumab was parallelly tested in our CRC model. Previous studies suggest *ARID1A* mutation promotes angiogenesis in liver cancer, supporting the potential of targeting tumor vessel formation as a treatment strategy^[^ ^52^ ^]^ *ARID1A/TP53* concurrent mutant SNUC5 xenografts were treated with vehicle, MK‐1775, bevacizumab, or the combination for 21 days (Figure S8A, Supporting Information). Notably, the combination therapy demonstrated the best efficacy without substantial weight loss (Figures S8B–D and S9F, Supporting Information). We observed reduced pCDK1 and Ki‐67 staining with a modestly increased γH2AX staining in the combination group, suggesting that the combination more effectively suppressed proliferation compared to the monotherapy (Figure S8E,F, Supporting Information). To assess tumor vascular activity in our models, we performed CD31 IHC staining in tumors from our xenografts. Both bevacizumab and its combination with MK‐1775 significantly reduced microvascular density in SNUC5 xenografts (Figure S8E,F, Supporting Information), which is detrimental to tumor growth.

These findings demonstrated that the patient with both *ARID1A/TP53* mutation showed a positive response to WEE1 inhibitor SC0191, as evidenced by both radiological and molecular improvements, and bevacizumab notably enhanced WEE1 inhibition efficacy as confirmed in our CRC models.

### 2.6 Co‐Targeting WEE1 and AKT is a Potential Combination Strategy in *ARID1A/TP53* Concurrent Mutant CRC

Given the limited efficacy of WEE1 inhibitor monotherapy observed in vivo, contrasted with the encouraging clinical responses in patients receiving WEE1 inhibition in combination with anti‐VEGF therapy, we aim to systematically explore rational co‐targeting strategies that could enhance and sustain the antitumor efficacy of WEE1 inhibition in this genetic context. Previous studies have also shown that WEE1‐inhibitor‐based combinations significantly enhanced response rates.^[^ ^28^, ^53^, ^54^ ^]^

**Figure 6:**
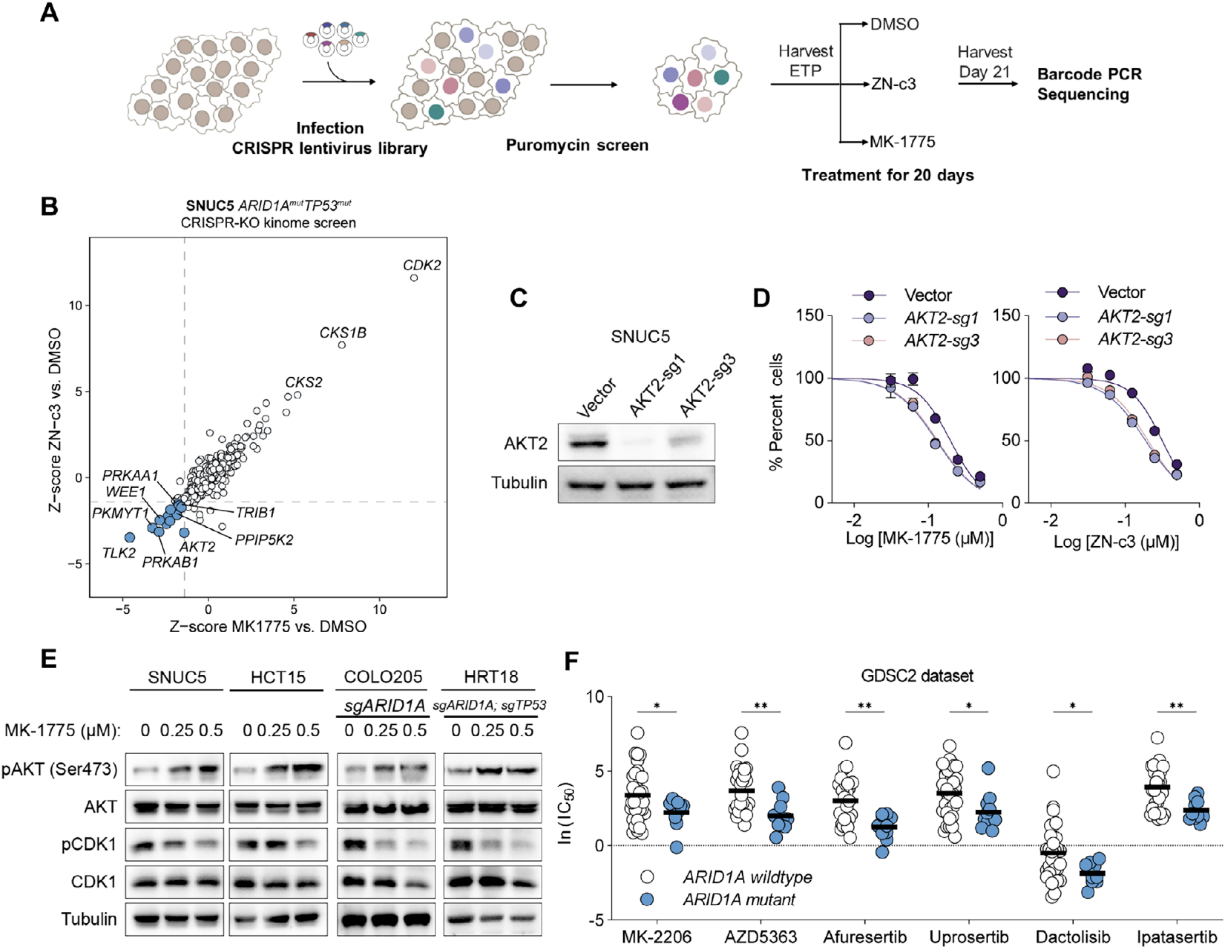
CRISPR screening revealed that co‐targeting WEE1 and AKT as a potential combination strategy in *ARID1A/TP53* concurrent mutant CRC. A) Schematic description of the CRISPR knockout kinome library screen. (B) Results of CRISPR screen in SNUC5 cells. Scatter plots present the Z‐scores of average log_2_(fold change) for MK‐1775 vs DMSO (*x*‐axis) and for ZN‐c3 vs DMSO (*y*‐axis). (C) Immunoblot analysis confirming AKT2 knockout in SNUC5 cells (n=2). (D) Dose‐response curves of SNUC5 cells, treated with MK‐1775 (left) or ZN‐c3 (right) for 5 days. The results are representative of three independent experiments, each done in triplicate. Bars represent mean ± SD. (E) Immunoblot analysis of HCT15, SNUC5, COLO205, and HRT18 cell lines treated with 0.25 or 0.5 µm MK‐1775 (n = 2). (F) GDSC2 analysis of indicated PI3K or AKT inhibitors according to *ARID1A* mutation status. Data are presented as scatter plots. Mean values are indicated by red lines. ^*^ *p* < 0.05, ^**^ *p* < 0.01 (two‐tailed Wilcoxon rank‐sum test).

To investigate additional combination candidates, we conducted a CRISPR‐KO screen (Brunello^[^ ^55^ ^]^) targeting 763 human kinase genes on SNUC5, an *ARID1A/TP53* mutant cell line. SNUC5 cells were infected with a CRISPR lentivirus library and selected by puromycin. The infected cells were then treated with MK‐1775, ZN‐c3, or DMSO for 20 days and harvested (**Figure** **6**A). The fold change between the treatment arms and DMSO were normalized with Z‐score (Figure 6B; Table S4, Supporting Information). The results showed a high correlation between the Z‐score of two WEE1 inhibitors. The depletion of *PKMYT1* (another inhibitory kinase targeting CDK1^[^ ^56^ ^]^) enhanced the efficacy of WEE1 inhibitors. Conversely, CDK2 depletion appeared to reduce the effectiveness of WEE1 inhibition (Figure 6B), which aligns with previous studies that Cyclin E‐CDK2 complex participates in cytotoxicity of WEE1 inhibition by promoting G1/S entry and replication stress.^[^ ^25^, ^57^, ^58^, ^59^ ^]^ These findings further validate the robustness of our CRISPR screen.

**Figure 7:**
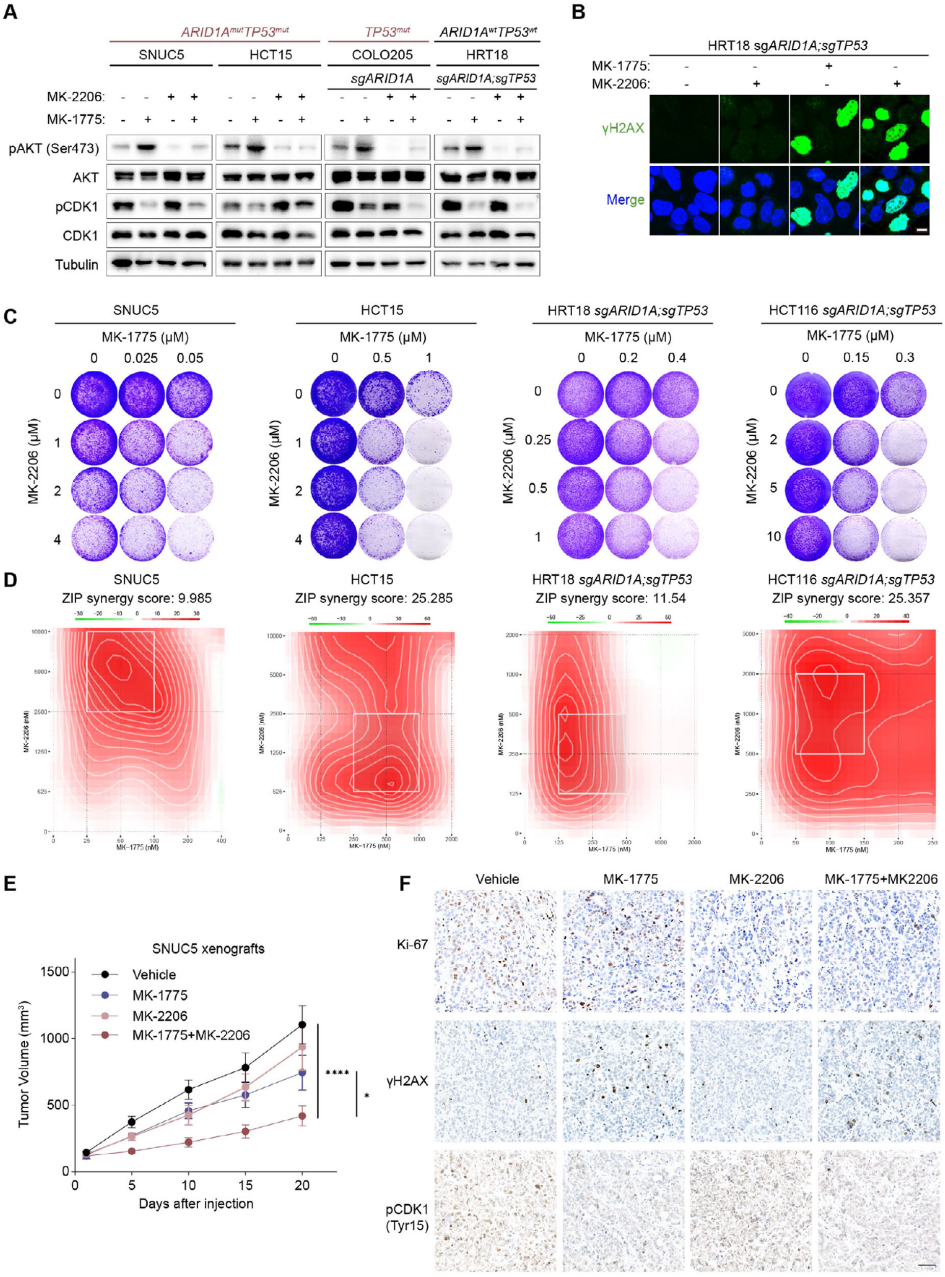
Preclinical efficacy of AKT and WEE1 co‐inhibition in *ARID1A/TP53* concurrent mutant CRC. A) Immunoblotting analysis of on‐target effect of MK‐2206 in HCT15 (*ARID1A/TP53* mutant), SNUC5 (*ARID1A/TP53* mutant), COLO205 (*TP53* mutant), and HRT18 cell lines. Cells were treated with 0.5 µm MK‐1775 and 2 µm MK‐2206, or a combination for 24 h (n=2). (B) Representative immunofluorescent images of HRT18 cells treated with 0.5 µm MK‐1775 and 2 µm MK‐2206, or combination for 24 h. Scale bar = 15 µm. (C) Crystal violet staining of SNUC5, HCT15, HRT18, and HCT116 cell lines treated with the indicated concentration of MK‐1775 or MK‐2206 alone or together for 7–10 weeks (n = 3). (D) The Synergistic effect of MK‐1775 and MK‐2206 combination was analyzed by SynergyFinder using the Zero Interaction Potency (ZIP) model. The inhibition rate was used to calculate ZIP synergy score, which indicates a synergistic effect when above 10. The box indicates the most synergistic area. (E) Tumor growth curves of SNUC5 xenografts treated with vehicle, MK‐1775 (60 mg kg^−1^), MK‐2206 (120 mg kg^−1^), or the combination (n = 5). MK‐1775 was administered via gavage daily, and MK‐2206 was administered three times a week. Data are shown as mean ± SEM; ^*^ *p* < 0.05, ^****^ *p* < 0.0001 (two‐way ANOVA test). (F) Representative immunohistochemical images of Ki‐67, γH2AX, and pCDK1 staining of the SNUC5 xenograft tumors. Scale bar = 100 µm.

We noticed that *AKT2* was one of the top hits driving resistance of both WEE1 inhibitors (Figure 6B). *AKT2* knockout sensitized SNUC5 to WEE1 inhibitors (Figure 6C,D), suggesting AKT inhibitors as a potential strategy for the combination with WEE1 inhibitors. Additionally, GDSC2 analysis indicated that several PI3K‐AKT inhibitors were preferably sensitive to *ARID1A*‐mutant CRC cells compared with wildtype cells, demonstrating PI3K‐AKT pathway as a potential therapeutic target for *ARID1A*‐mutant CRC (Figure 6F). Interestingly, only in cells with a concurrent mutation background, WEE1 inhibition led to a dose‐dependent increase in AKT phosphorylation (Figure 6E; Figure S9A, Supporting Information). To further investigate this, we tested whether AKT inhibitor (MK‐2206) could enhance the efficacy of the WEE1 inhibitor in our CRC models with *ARID1A/TP53* deficiency. We found that MK‐2206 effectively inhibited phosphorylation of AKT (**Figure** **7**A). Notably, the combination treatment induced a dose‐dependent enhancement in both growth inhibition and DNA damage accumulation compared with either monotherapy (Figure 7B,C; Figure S9B,C, Supporting Information). Furthermore, the Zero Interaction Potency (ZIP) synergy model demonstrated that the combinations exhibited synergistic effects, with the synergy score roughly above 10 (Figure 7D; Figure S9D, Supporting Information).

Next, we evaluated the therapeutic efficacy of the combination therapy in SNUC5 xenografts. Mice were treated with vehicle, MK‐1775, MK‐2206, or combinations of MK‐1775 and MK‐2206 for 20 days (Figure S10A, Supporting Information). Notably, both monotherapy and combination therapy suppressed tumor growth without causing significant weight loss, with the combination therapy demonstrating the best efficacy (Figure 7E; Figure S10B–D, Supporting Information). Immunohistochemical analysis revealed significantly weaker pCDK1 and Ki‐67 staining in the MK‐1775 and MK‐2206 combination group, indicating that the combination therapy more effectively inhibited tumor cell proliferation compared to either monotherapy (Figure 7F; Figure S10E, Supporting Information). These results suggest that targeting both the AKT and WEE1 pathways could be an effective therapeutic strategy for *ARID1A*‐mutant CRC.

Our data demonstrate that WEE1 inhibition leads to AKT activation. Prior studies have indicated that AKT pathway activation suppresses R‐loop accumulation by recruiting the helicase *DHX9*.^[^ ^60^ ^]^ Given our previous finding that R‐loop levels are a key determinant of WEE1 inhibitor sensitivity, we hypothesized that AKT inhibition would enhance WEE1 inhibitor efficacy by promoting R‐loop accumulation. To test this, we assessed the R‐loop in *ARID1A/TP53* concurrent mutant SNUC5 cells under AKT blockade. As expected, SNUC5 cells inherently exhibited high R‐loop levels, consistent with our previous findings. However, neither *AKT2* knockout nor pharmacologic AKT inhibition resulted in any significant alteration of R‐loop levels or *DHX9* expression (Figure S10F–H, Supporting Information). These findings suggest that AKT inhibition enhances WEE1 inhibitor‐induced DNA damage and growth suppression through an R‐loop‐independent mechanism, which warrants further investigation.

## 3 Discussion

*ARID1A* mutations are the most frequent alterations in the SWI/SNF complex in CRC, representing a distinct subset of genetically unstable tumors.^[^ ^14^, ^15^, ^16^ ^]^ As a key subunit of the SWI/SNF complex, *ARID1A* plays a crucial role in regulating chromatin accessibility, participating in a wide range of cellular pathways, including the extensively studied DDR pathway in recent years.^[^ ^61^ ^]^ Typically, *ARID1A* mutations are often truncating mutations,^[^ ^12^, ^14^ ^]^ suggesting its function as a tumor suppressor, which might be a challenging target for direct drug development. Exploiting synthetic lethality offers a promising strategy to target such mutations.^[^ ^8^, ^61^, ^62^, ^63^ ^]^ While several studies suggest that *ARID1A*‐mutant tumors might be vulnerable to DDR modulators and immunotherapy,^[^ ^12^, ^16^, ^18^ ^]^ these findings are mostly confined to preclinical models, highlighting the need for more practical therapeutic strategies.

In our present study, we identified WEE1 kinase as a vulnerability in *ARID1A*‐mutant CRC. *ARID1A* plays multifaceted roles in the context of DNA damage repair. Broadly, its functions can be categorized into two main aspects: First, as a chromatin remodeler, *ARID1A* contributes to genome stability by preventing DNA damage formation. Studies have demonstrated that *ARID1A* suppresses genome‐wide R‐loop accumulation, thereby mitigating replication stress and associated DNA damage.^[^ ^12^, ^18^, ^19^, ^64^ ^]^ Second, *ARID1A* functions as a signaling regulator involved in the coordination of DNA damage repair pathways. It has been reported that *ARID1A* is recruited to DNA break sites, where it sustains ATR activation and HR activity to facilitate efficient DNA repair.^[^ ^17^, ^20^ ^]^ Intriguingly, our experimental data specified that *ARID1A* mutation alone was insufficient to make CRC vulnerable to WEE1 inhibition.

Instead, *ARID1A*/*TP53* concurrent mutations might be a better predictive biomarker for WEE1 inhibitors, which occur in ≈4% of all colorectal cancers.^[^ ^10^ ^]^ In our SC0191‐based clinical trial, we found patients with concurrent mutations, rather than either *ARID1A* or *TP53* mutation alone, that lead to robust response to WEE1 inhibition. Additionally, an unpublished clinical trial conducted in our center testing WEE1 inhibitor as monotherapy also indicated that three mCRC patients harbored with *ARID1A/TP53* concurrent mutations had longer progression‐free survival (PFS) than others. However, these clinical studies were not biomarker‐driven and were limited by a small sample size, which should be considered as limitation. Further clinical trials with larger sample sizes are needed to validate whether co‐mutational status confers an enhanced response to WEE1 inhibition.

Mechanistically, tumor cells are typically guarded by G1/S and G2/M checkpoints during the cell cycle, which are under regulation by P53 and WEE1, respectively. Previous studies suggest that tumors with *TP53* mutations are more reliant on G2/M checkpoints regulated by WEE1, which leads to synthetic lethality when being inhibited.^[^ ^33^, ^65^ ^]^ However, *TP53* mutation alone has not been a clear biomarker for predicting responses to WEE1 inhibitors according to several clinical trials.^[^ ^25^, ^66^ ^]^ We propose that the escape of both G1/S and G2/M checkpoints due to *TP53* mutations and WEE1 blockade alone is not sufficient to induce lethal DNA damage in tumor cells. Instead, additional drivers of DNA damage, such as replication stress, are also crucial.

Here, our current study demonstrated that *ARID1A* knockout promoted the accumulation of DNA‐RNA hybrids (R‐loops) and replication stress, providing substantial sources of DNA damage, which in turn rendered *TP53*‐mutant CRC cells responsive to WEE1 inhibition. Besides, ATAC‐seq analysis revealed widespread chromatin changes following *ARID1A* knockout, indicating that *ARID1A* may also regulate DDR gene expression at the transcriptional level. We identified *ATF3* as a transcription factor critically involved in regulating the DDR pathway. *ARID1A* knockout led to chromatin closure of *ATF3*, resulting in suppression of the P53 and UV response pathways. Previous studies have reported that ATF3 is activated by P53 and can interact with Tip60 to promote DNA damage sensing and activate the ATM pathway^[^ ^50^ ^]^ Repression of *ATF3*, after UV‐mediated genotoxic stress, impairs the DNA repair process^[^ ^51^ ^]^ Taken together, in *ARID1A* and *TP53* dual deficient model, R‐loop accumulation promotes DNA damage, while *ATF3* suppression impairs DNA repair, collectively leading to substantial DNA damage accumulation that underlies the therapeutic vulnerability to WEE1 inhibition.

In high‐grade serous ovarian cancer, which is characterized by high genomic instability and alterations in cell cycle,^[^ ^67^, ^68^ ^]^ MK‐1775 monotherapy had reached over 20% Objective Response Rate (ORR) from these patients.^[^ ^26^ ^]^ While in patients with mCRC who had received MK‐1775 monotherapy, the ORR was less than 2%.^[^ ^29^ ^]^ Currently, WEE1 inhibitor‐based combination strategies are under active exploration, including chemotherapy and radiotherapy combinations, which significantly enhance patient responses.^[^ ^53^, ^69^, ^70^ ^]^ However, it is often observed that enhanced responses tend to be accompanied by poorer tolerance and a higher incidence of adverse effects. In the context of our clinical research, we identified a well‐tolerated and effective strategy of combining WEE1 inhibitor and bevacizumab in patients with concurrent *ARID1A* and *TP53* mutations. This finding was further corroborated in our xenograft models. The efficacy of bevacizumab in *ARID1A*‐mutant tumors was possibly attributed to the angiogenesis effect of *ARID1A* mutation.^[^ ^52^ ^]^

Evidence that bevacizumab enhances the efficacy of WEE1 inhibition indicates that the therapeutic potential of WEE1 inhibitors can be further augmented through rational combination strategies. To systematically screen combination candidates of WEE1 inhibitors in tumor cells, we performed a CRISPR knockout screening, which identified *AKT2* as one of the top hits among 762 human kinase genes. Previous studies have reported a robust synergistic effect of co‐inhibiting WEE1 and the PI3K‐AKT‐mTOR pathway using PI3K or mTOR inhibitors,^[^ ^71^, ^72^, ^73^, ^74^ ^]^ which highlights the strong crosstalk between these pathways and prompted us to investigate the combination of WEE1 and AKT inhibitors.

The therapeutic efficacy of combined WEE1 and AKT inhibition in *ARID1A/TP53* concurrent mutant models may involve several mechanisms. First, *ARID1A*‐deficient tumors, especially bladder cancers, are intrinsically dependent on the PI3K‐AKT rather than the MAPK pathway for proliferation and survival, and consequently exhibit heightened sensitivity to inhibition of the PI3K‐AKT axis.^[^ ^75^, ^76^ ^]^ Second, AKT inhibition induces replication stress and suppresses DNA damage repair pathways.^[^ ^77^, ^78^ ^]^ Interestingly, we observed AKT activation following WEE1 inhibition, suggesting that AKT upregulation may engage DDR signaling and thereby attenuate the efficacy of WEE1 blockade. Consistent with this notion, combined AKT and WEE1 inhibition further increased DNA damage accumulation. Previous studies in ovarian cancer models have shown that AKT activation alleviates replication stress in a DHX9‐dependent manner by resolving R‐loops.^[^ ^60^ ^]^ However, given the distinct biological context of colorectal cancer, we did not detect notable changes in R‐loop upon AKT blockade, indicating that the enhanced cytotoxicity of the combination therapy operates through an R‐loop‐independent way. Alternatively, AKT inhibition has been reported to suppress *ATF3* expression,^[^ ^79^ ^]^ which may in turn weaken DNA repair and sensitize cells to WEE1 inhibition, though this remains to be further explored.

Together, our study elucidates the mechanism underlying the sensitivity of *ARID1A/TP53* concurrent mutant CRC to WEE1 inhibition. We demonstrate that *ARID1A* loss promotes DNA damage through two mechanisms: increased replication stress due to R‐loop accumulation and weakened DNA damage repair caused by downregulation of *ATF3*. WEE1 inhibition and *TP53* mutation together disrupt the G1/S and G2/M cell cycle checkpoints, forcing cells to accumulate the unrepaired DNA damage stemming from *ARID1A* loss, ultimately triggering cell death. We further show an adaptive response of AKT activation to the WEE1 inhibitor. AKT inhibition enhances WEE1 inhibitor efficacy through a non‐R‐loop‐dependent manner, thereby revealing a promising combination strategy. These findings highlight the need for future clinical studies evaluating this combination in patients with *ARID1A/TP53* concurrent mutations.

## 4 Experimental Section

### Data and Code Availability

The cell line drug screening and dependency data are available in a public, open‐access repository. CRISPR dependency and mutation data are downloaded from DepMap (https://depmap.org/portal/data_page/?tab=allData). GDSC2 drug sensitivity data are obtained from the R package “oncoPredict^[^ ^80^ ^]^”. Bulk RNA sequencing, ATAC sequencing, and CRISPR screening raw data are available at Genome Sequence Archive for Human (BioProject: PRJCA047507; Accession ID: HRA013748). The WES data is not publicly available due to patient privacy considerations, but is available upon reasonable request to the corresponding authors.

- This paper does not report the original code.
- Any additional information required in this paper is available from the lead contact upon request.

### Cell Lines and Cell Culture

All cell lines were derived from the Stem Cell Bank, Chinese Academy of Sciences between September 2022 and June 2023, which were grown at 37 °C with 5% CO_2_ (CO_2_ free for L15‐based cell culture). COLO205 (RRID: CVCL_F402), HRT18 (RRID: CVCL_2514), HCT15 (RRID: CVCL_0292), LS180 (CVCL_0397), and SNUC5 (RRID: CVCL_5112) cells were cultured in RPMI 1640 (BasalMedia, #L210KJ) with 10% FBS (Sigma) and 1% penicillin‐streptomycin (ThermoFisher, #15140122). SW48 (RRID: CVCL_1724), LOVO (RRID: CVCL_0399), and RKO (RRID: CVCL_0504) cells were cultured in DMEM (BasalMedia, #L110KJ) with 10% FBS and 1% penicillin‐streptomycin. SW620 (RRID: CVCL_0547), and SW1116 (RRID: CVCL_0544) were grown in L15 (BasalMedia, #L620KJ) with 10% FBS and 1% penicillin‐streptomycin. HCT116 (RRID: CVCL_0291) was grown in McCoy's 5A (BasalMedia, #L630KJ) with 10% FBS (Sigma) and 1% penicillin‐streptomycin. All cell lines were tested negative for mycoplasma contamination regularly using Myco‐Lumi (Beyotime, #C0297S).

### Genetic Constructs and Lentiviral Production

Lentivirus‐based sgRNAs (lentiCRISPRv2 vector) were obtained from Shanghai Genechem. The sgRNA sequences were listed in Table S5 (Supporting Information). cDNA sequence encoding full length RNASEH1 was cloned into pcDNA3.1(‐) and was used for overexpression. The lentiviruses were generated using standard protocols.^[^ ^81^ ^]^ For lentiviral infection of cells grown in a 6‐cm dish, cells were transduced with 3 mL of viral particle‐containing medium with 8–10 µg mL^−1^ polybrene. After incubation for 10 h, the medium was replaced. After 2 days, the infected cells were selected with puromycin, hygromycin, or G418 for 5–7 days. Protein lysates were collected to confirm knockout or overexpression efficacy by immunoblotting.

### siRNA and Plasmid Transfections

cDNA, siRNAs for *ATF3*, and negative control siRNA were obtained from Shanghai Genechem. Cells were transfected using Lipofectamine 3000 Transfection Reagent (Invitrogen, Waltham, MA, USA) according to the manufacturer's instructions. Gene expression was confirmed by western blotting.

### Cell Proliferation Assays

≈1000 cells were seeded in 96‐well plates and incubated overnight. The cells were treated with the indicated compounds for the indicated number of days. Cell viability was detected using the CellTiter‐Glo (Promega, #G7570) according to the manufacturer's protocol. The percentage of cell viability was calculated relative to that of the untreated control or baseline cells. Combined effects were analyzed using SynergyFinder (https://synergyfinder.org/).

### Antibodies and Drugs

For details, see Table S6 and S7 (Supporting Information).

### Crystal Violet Staining

≈5 × 10^3^ cells/well (for HCT15), 2 × 10^4^ cells/well (for HCT116 and HRT18), or 5 × 10^4^ cells/well (for SW620 and SNUC5) were seeded in six‐well plates with the indicated treatment after 24 h. The culture media/drugs were refreshed every 3‐4 days. When control wells were confluent, colonies were rinsed with PBS, fixed with 4% paraformaldehyde for 15 min, and stained with crystal violet solution (Beyotime, #C012) for 10 min. The crystal violet solution was discarded, and plates were rinsed twice with H_2_O and air‐dried.

### Immunofluorescence

Cells were plated on 35 mm confocal dishes (Biosharp, #BS‐20‐GJM) and incubated overnight for attachment. After the indicated treatments, the cells were fixed for 10 min at room temperature in 4% PFA and permeated with 0.5% Triton X‐100 for 15 min. The primary antibodies were incubated overnight at 4 °C. Secondary antibodies and DAPI solution (Beyotime, #C1005) were incubated for 1.5 h and 10 min, respectively at room temperature. Images were acquired using an Olympus FV3000 confocal microscope, and analysis was performed using ImageJ v2.0.0.

### Immunoblotting

Cell pellets were lyzed with RIPA buffer with protease/phosphatase inhibitor cocktail for 20 min on ice. The cell lysis was centrifuged at 13000 g for 15 min at 4 °C, and the supernatant was harvested. Protein extracts were diluted in 4× SDS loading buffer and boiled for 10 min at 97 °C before separation by SDS‐PAGE. Proteins were transferred to nitrocellulose membranes (Cytiva, #10600001), then blocked with 5% non‐fat milk in TBST. The nitrocellulose membranes were incubated overnight with primary antibodies and next probed with HRP‐conjugated secondary antibodies at room temperature for 1.5 h. The image was captured by the Tanon 5200 system.

### EdU Incorporation and Detection

Incubate the cells with pre‐warmed culture medium containing 10 µm EdU for 2 h. Remove the medium and add 1 mL of fixation solution. Fix the cells at room temperature for 15 min. Remove the fixation solution, wash the cells, and permeabilize with 0.3% Triton X‐100 at room temperature for 15 min. After washing, use the BeyoClick EdU assay kit (Beyotime, #C0078S): add 0.5 mL of Click reaction solution, gently shake, and incubate for 30 min at room temperature in the dark. Finally, nuclei were stained with DAPI and mounted. Images were acquired with an Olympus FV3000 confocal microscope. The EdU entire population nuclear intensity and percentage of cells incorporating EdU were determined.

### Flow Cytometry

Cells were synchronized at the G1/S transition using a 24‐h incubation with 2.5 mm thymidine. After extensive washing, cells were released and harvested at indicated time points and subsequently fixed in ice‐cold 70% ethanol overnight at 4 °C. Fixed cells were washed with PBS and resuspended in PBS with propidium iodide (PI; 20 µg mL^−1^) and RNase A (200 µg mL^−1^), incubated at 37 °C for 40 min and finally measured on the flow cytometer. FlowJo software was used to analyze data.

### RNA‐Seq Sample Preparation, Sequencing, and Analysis

Cells were seeded in T25 flasks (1 × 10^6^ COLO205 vector and COLO205 sg*ARID1A*) and treated with DMSO, 0.5 µm MK‐1775, or 0.5 µm ZN‐c3 in triplicate for 48 h. For HRT18 cells, 1 × 10^6^ *TP53^KO^* and *ARID1A^KO^/TP53^KO^* cells were collected for sequencing. Total RNA was extracted by Qiagen RNeasy kit. The TruSeq RNA sample preparation kit was used for the mRNA purification, fragmentation, the first‐strand and the second‐strand cDNA synthesis. The libraries were sequenced on NovaSeq XPlus (Illumina) at a depth of ≈20 m reads per sample. Heatmaps were generated using the R package “pheatmap”. Differential expression analysis was performed using the R package “DESeq2”. For COLO205 cells, GO enrichment (molecular function) was analyzed with differentially expressed genes filtered with log2 (fold change) > 0 and FDR < 0.05. For HRT18 cells, GSEA enrichment was performed based on hallmark gene sets.

### ATAC‐Seq Sample Preparation, Sequencing and Analysis

ATAC‐seq was performed by Hyperactive ATAC‐Seq Library Prep Kit (Vazyme, #TD711‐01) according to manufacturer's protocol. For library preparation, organoids were dissociated into single cells. 50000 cells were resuspended in 50 µL of ATAC‐seq lysis buffer (10 mm Tris‐HCl pH 7.4, 10 mm NaCl, and 3 mm MgCl2 in distilled water) containing 0.1% NP40, and were incubated on ice for 10 min. After lysis, the lysate was centrifuged at 500 g for 5 min at 4 °C. Supernatant was removed, and nuclei were resuspended in 50 µL of transposition mix (10 µL 5X TTBL buffer, 5 µL Tn5 transposase, 35 µL ddH2O) and incubated for 30 min at 37 °C. Transposed DNA was purified using VAHTS DNA Clean Beads, and subsequently amplified with sequencing primers and PCR mix for 10–15 cycles. PCR‐amplified DNA was purified using VAHTS DNA Clean Beads and sequenced on an Illumina HiSeq X with 150 bp pair‐end reads. Each sample generated ≈20‐40 million mapped reads for the next analysis. Heatmaps were generated using the Linux software “deeptools”. Differential peak analysis and annotation were performed using the R package “DiffBind” and “ChIPseeker”.

### Alkaline Comet Assay

A Comet Assay Kit (catalog 4250‐050‐K; Trevigen) was used to perform the alkaline comet assay according to the manufacturer's instructions. Briefly, a 5 µL volume of cells at 1 × 10^5^ mL^−1^ was added to 50 µL molten LMAgarose (at 37 °C), and 50 µL of the mixture was immediately pipetted onto a comet slide. After the mixture of agarose and cells was evenly dispersed, the slides were placed flat at 4 °C in the dark for 30 min in a high‐humidity environment. The cells were then lyzed overnight by immersing slides in lysis buffer. After lysis, the excess buffer was drained from the slides, and the slides were immersed in freshly prepared alkaline unwinding solution, pH >13, for 1 h at 4 °C in the dark before application of an electric field. The electrophoresis was performed under ice. An electric field (21 V) was applied to the cells for 40 min at 4 °C, and the cells were stained with SYBR gold (catalog S11494; Thermo Fisher Scientific) for 30 min in the dark and photographed using an Olympus microscope with an attached camera. The comets were analyzed using ImageJ.

### CRISPR Knockout Library Titration

Accurate virus volumes to be used in the screen were determined in SNUC5 cell line to achieve 30% to 50% infection rate. Human kinome CRISPR knockout library (Brunello) was obtained from Addgene (#73179) and was packaged into lentivirus. Spinfection was performed in 12‐well plates containing 1‐2 × 10^6^ cells per well, with different virus volumes (0, 20, 40, 60, 80, 100 µL), and a final concentration of 8 µg mL^−1^ polybrene. Cells were spinfected for 1 h at 600 g, 29 °C, and then incubated at 37 °C for 6–8 h. The infected cells were then collected and seeded into duplicate wells of six‐well plates. Two days after seeding, the medium was changed, one well as a control, and another well treated with 1 µg mL^−1^ puromycin for another 2 days. After puromycin selection, the cells in the control and puromycin‐selected groups were counted. The virus volume and cell amount in 12‐well plates for the spinfection that yielded 30% to 50% infection efficiency, as inferred by survival with puromycin selection, were used for large‐scale screening.

### CRISPR Knockout Screens

SNUC5 cells were infected with the lentiviral sgRNA library (Brunello) at a low MOI (≈0.3), and medium containing 1 µg/ml puromycin (InvivoGen, #ant‐pr‐1) was added on the 5^th^ day to select the infected cells. Three days after selection, cells were pooled together and divided into three groups, including DMSO, 0.1 µm MK‐1775, and 0.1 µm ZN‐c3 treatment groups. Cells were cultured for 20 days and were passaged every 3–5 days. An average representation of >1500 cells per clone were maintained during the culture. Genomic DNA was isolated using the DNeasy Blood & Tissue Kit (Qiagen, #69504), and genome‐integrated sgRNA sequences were amplified by PCR using Ex Taq DNA polymerase (Clontech, RR001A). i5 and i7 multiplexing barcodes were added in a second round of PCR, and final gel‐purified products were sequenced on an Illumina Nova‐X‐Plus system to determine sgRNA representation in each sample. Sample data analysis was performed using MAGeCK software previously described https://sourceforge.net/projects/mageck.

### Animal Studies

The experimental procedures on mice were handled according to the guidelines of the Ethical Committee of Fudan University (IDM2023039). The BALb/c nude mice (male, 6 weeks old) were purchased from Charles River Laboratories. ≈5 × 10^5^ COLO205 cells or 1 × 10^6^ SNUC5 cells were injected into the flank of the mice. When the mean tumor volume reached the indicated volume, mice were treated with the indicated drugs. Tumor size was measured with a caliper every 3‐5 days after treatment, and the tumor volume was calculated with the equation: V(mm^3^) = a × b^2^/2, where a is the longest diameter and b is the shortest diameter. Mice were sacrificed for tumor dissection when the tumor volume of the vehicle group reached the ethical endpoint. For drug preparation, MK‐1775 was suspended in 0.5% w/v CMC‐Na, and MK‐2206 was dissolved in 20% w/v sulfobutylether‐β‐cyclodextrin. Bevacizumab was dissolved in 0.9% NaCl and was infused via the tail vein.

### Immunohistochemistry

Formalin‐fixed cell line mouse subcutaneous grafts were dehydrated, paraffin‐embedded, and sectioned into 4 mm thick slices for immunohistochemical analysis. The sections were treated with primary antibodies overnight at 4 °C: For details, see Table S5 (Supporting Information). Next, sections were treated with secondary antibodies (#AFIHC001). The cell nucleus was stained with Mayer's Hematoxylin (#AFIHC005). The staining results were evaluated by an experienced pathologist.

### Whole‐Exome Sequencing (WES)

Peripheral blood and formalin‐fixed paraffin‐embedded (FFPE) tumor biopsy samples were prepared for WES detection. Peripheral blood mononuclear cells served as normal controls for filtering germline mutations. All the samples were shipped to Novogene to undergo further sequencing by Illumina platforms (PE150).

### Patient Ethics Statement

This study involves human participants and was approved by Zhongshan Hospital Ethics Committee (Approval No. B2024‐094R). Participants gave informed consent to participate in the study before taking part. Clinical trial information: NCT06363552.

### Statistical Analysis

Data are presented as mean ± S.D. or S.E.M., as indicated. The number of independent biological experiments for each experiment is noted in the figure legends. Statistical analysis was performed using Prism 10.4.0 (GraphPad). Comparisons between groups were performed using the Wilcoxon rank‐sum test or one‐way/two‐way ANOVA with post‐hoc tests (for >2 groups), as appropriate. *P*‐values are denoted by ^*^ *P* < 0.05, ^**^ *P* < 0.01, ^***^ *P* < 0.001, ^****^ *P* < 0.0001. The sample sizes and animal numbers were determined from pilot laboratory experiments and previously published studies. All statistical details of the experiments are mentioned in the figure legend.

## Conflict of Interest

The authors declare no conflict of interest.

## Supporting information



Supporting Information

Supplemental Table 1

Supplemental Table 2

Supplemental Table 3

Supplemental Table 4

Supplemental Table 5

Supplemental Table 6

## Data Availability

The data that support the findings of this study are available from the corresponding author upon reasonable request.
