# Supplementary material for: Targeting WEE1 in ARID1A/TP53 Concurrent Mutant Colorectal Cancer by Exploiting R‐Loop Accumulation and DNA Repair Deficiencies
Source: Adv Sci (Weinh). 2025 Nov 30;13(9):e12074. doi: 10.1002/advs.202512074 (PMC12904052; doi:10.1002/advs.202512074)
Supplement: Supplementary file 1 — Supporting Information [file ADVS-13-e12074-s006.pdf]

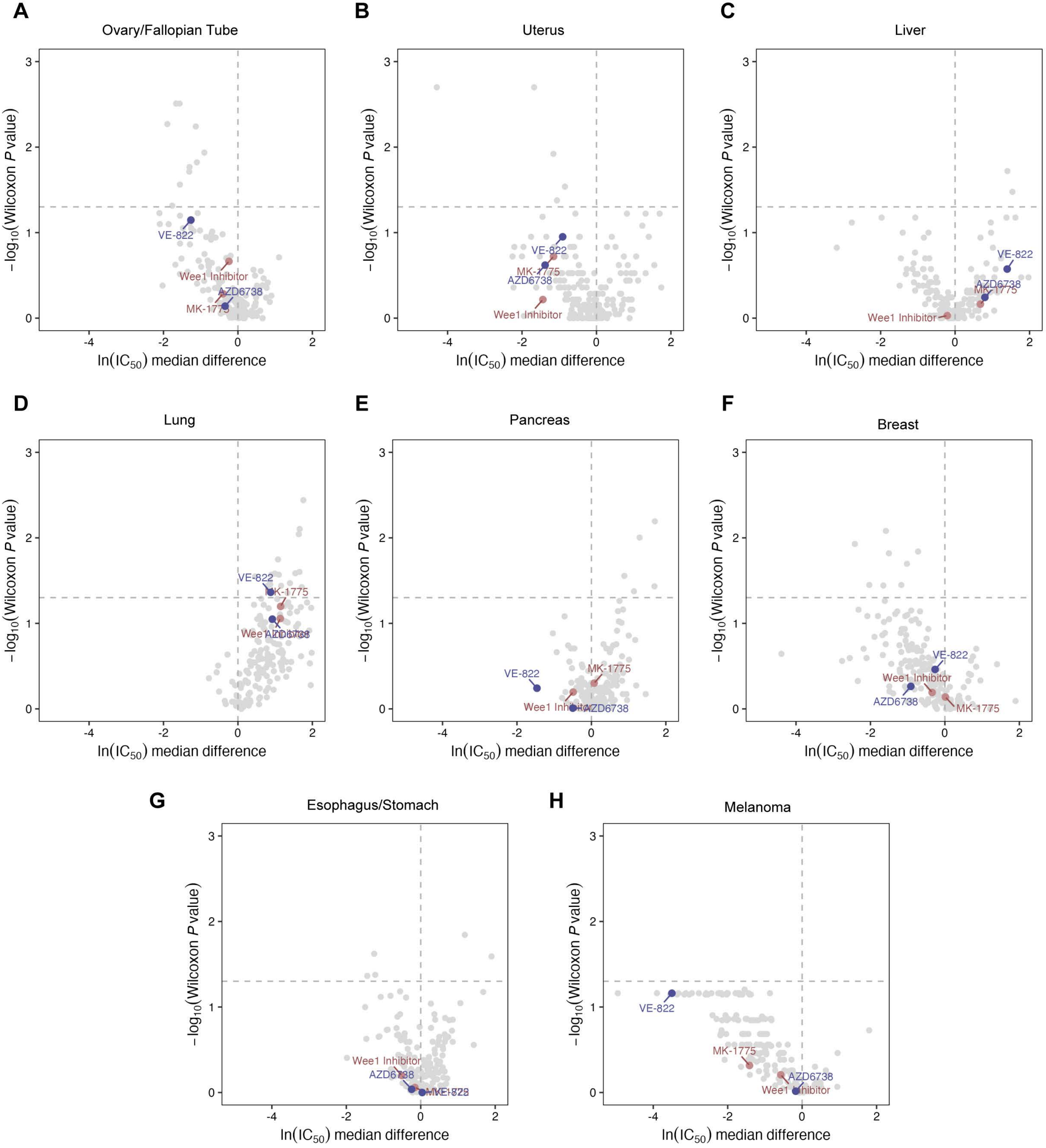

Figure S1. (**A-H**) Analysis of median difference of compounds (n=185) IC_{50} from the GDSC2 project grouped according to their *ARID1A* mutation status across different tumor types, including ovary/fallopian tube (**A**), uterus (**B**), liver (**C**), lung (**D**), pancreas (**E**), breast (**F**), esophagus/stomach (**G**) cancers, and melanoma (**H**). WEE1 inhibitors (MK-1775 and 681640) and ATR inhibitors (VE-822 and AZD-6738) are marked in each volcano plot.

A

CRC Cell lines	*TP53* mutation status	*ARID1A* mutation status
SNUC5	**R248W, V218L**	**Q766Sfs*67, W1073Mfs*32**
HCT15	**S241F, X367 splice**	**R270H**
COLO205	**Y107Ter**	/
SW620	**R273H, P309S**	/
SW1116	**A159D**	/
RKO	/	**D1850Tfs*33, P1115Qfs*46**
LOVO	/	**F2141Sfs*59**
SW48	/	**M1564***
HRT18	/	/
HCT116	/	/

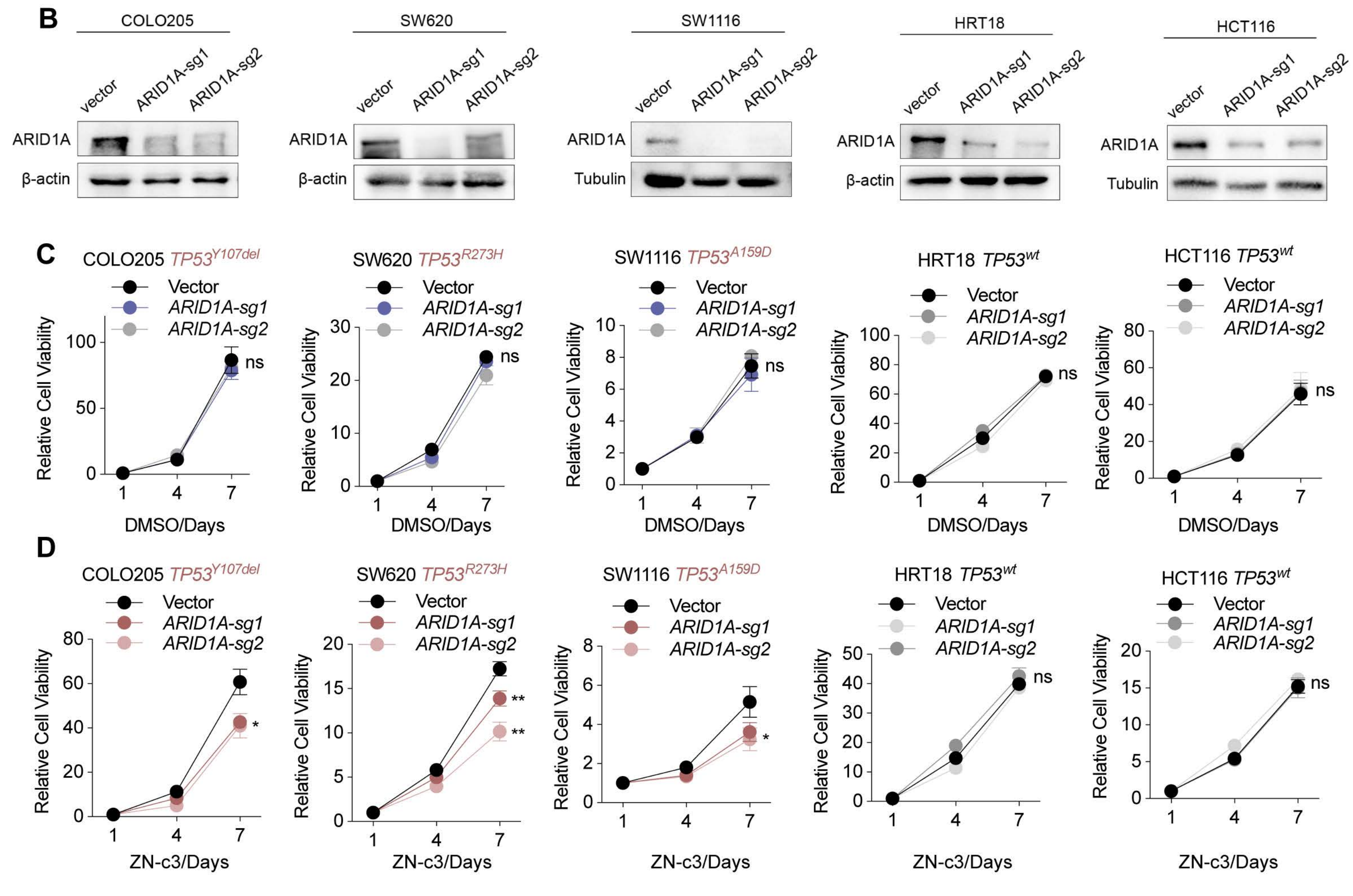

Figure S2. (**A**) *TP53* and *ARID1A* mutation information in different colorectal cancer cells according to DepMap cell line annotation.(**B**) Knockout efficacy of *ARID1A* sgRNA in the indicated cell lines (n = 2). (**C**) Cell proliferation assay of *ARID1A* knockout or vector cells for the indicated days. (**D**) Cell proliferation assay of *ARID1A* knockout or vector cells treated with 0.25 μM ZN-c3 for indicated days. The results are the representative of three independent experiments, each done in triplicate. Data are shown as mean ± SD. ns, not significant;*p<0.05, **p<0.01 (two-way ANOVA test).

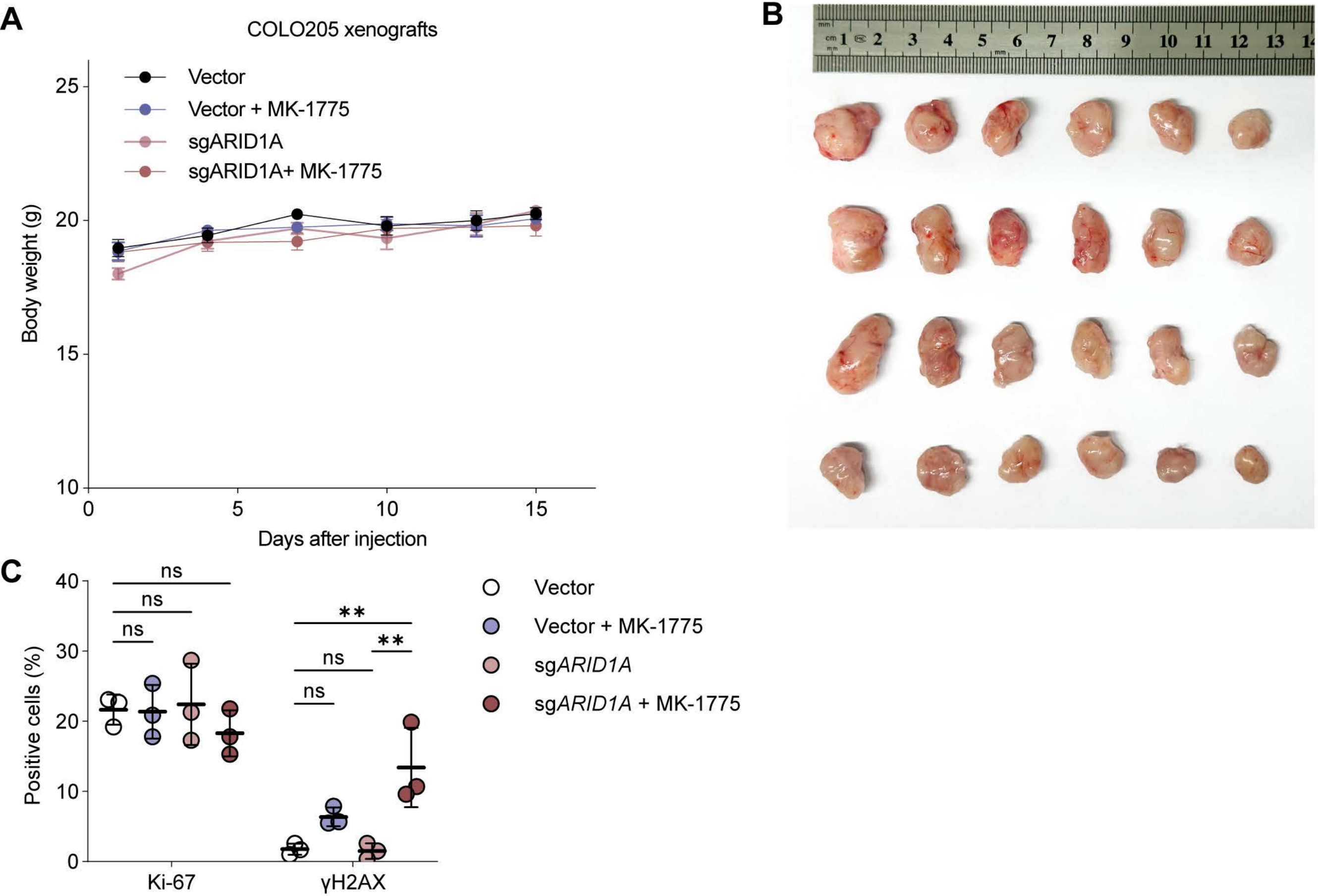

Figure S3. (**A**) Percentage body weight changes in COLO205 xenografts treated with vehicle or MK-1775 (60 mg/kg). (**B**) A broad view of COLO205 xenocraft tumors after euthanizing the mice at endpoint. (**C**) Quantification analysis of IHC results in Figure 1H (n = 3). Data are shown as mean ± SD. ns, not significant; **$p<0.01$ (two-way ANOVA test).

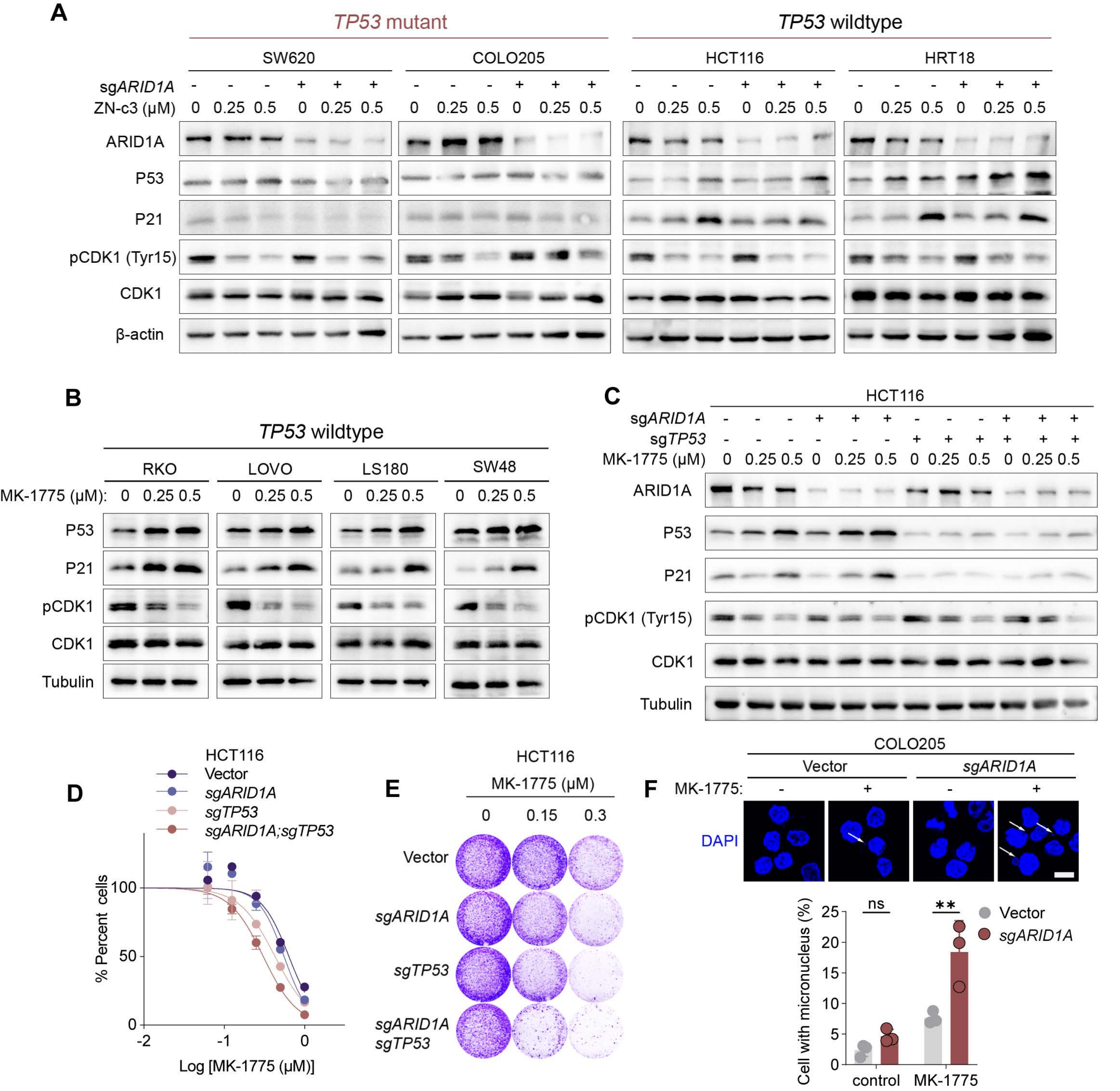

Figure S4. (A-B) Immunoblot analysis of ARID1A knockout (sg1) CRC cell lines treated with WEE1 inhibitors. COLO205 (TP53-mutant), SW620 (TP53-mutant), HCT116 (TP53 wildtype), HRT18 (TP53 wildtype), RKO (TP53 wildtype), LOVO (TP53 wildtype), LS180 (TP53 wildtype), and SW48 (TP53 wildtype) cells were treated with MK-1775 or ZN-c3 at the indicated concentrations for 48 h (n = 2). **(C)** Immunoblot analysis of TP53 and/or ARID1A knockout cells. HCT116 cells were treated with MK-1775 at the indicated concentrations for 48 h (n = 2). **(D)** Dose-response curves of HCT116 treated with MK-1775 for 5 days. The results are the representative of three independent experiments, each done in triplicate. Data are mean ± SD. **(E)** Crystal violet staining of HCT116 cells, treated with MK-1775 at the indicated concentration for 7-10 days (n = 3). **(F)** Representative immunofluorescent images of COLO205 cells after 0.5 μM MK-1775 treatment for 24 h (n = 3). Scale bar = 15 μm. The bottom panel shows the proportion of micronucleated cells. Data was shown as mean ± SD; ns, not significant; **p<0.001 (one-way ANOVA).

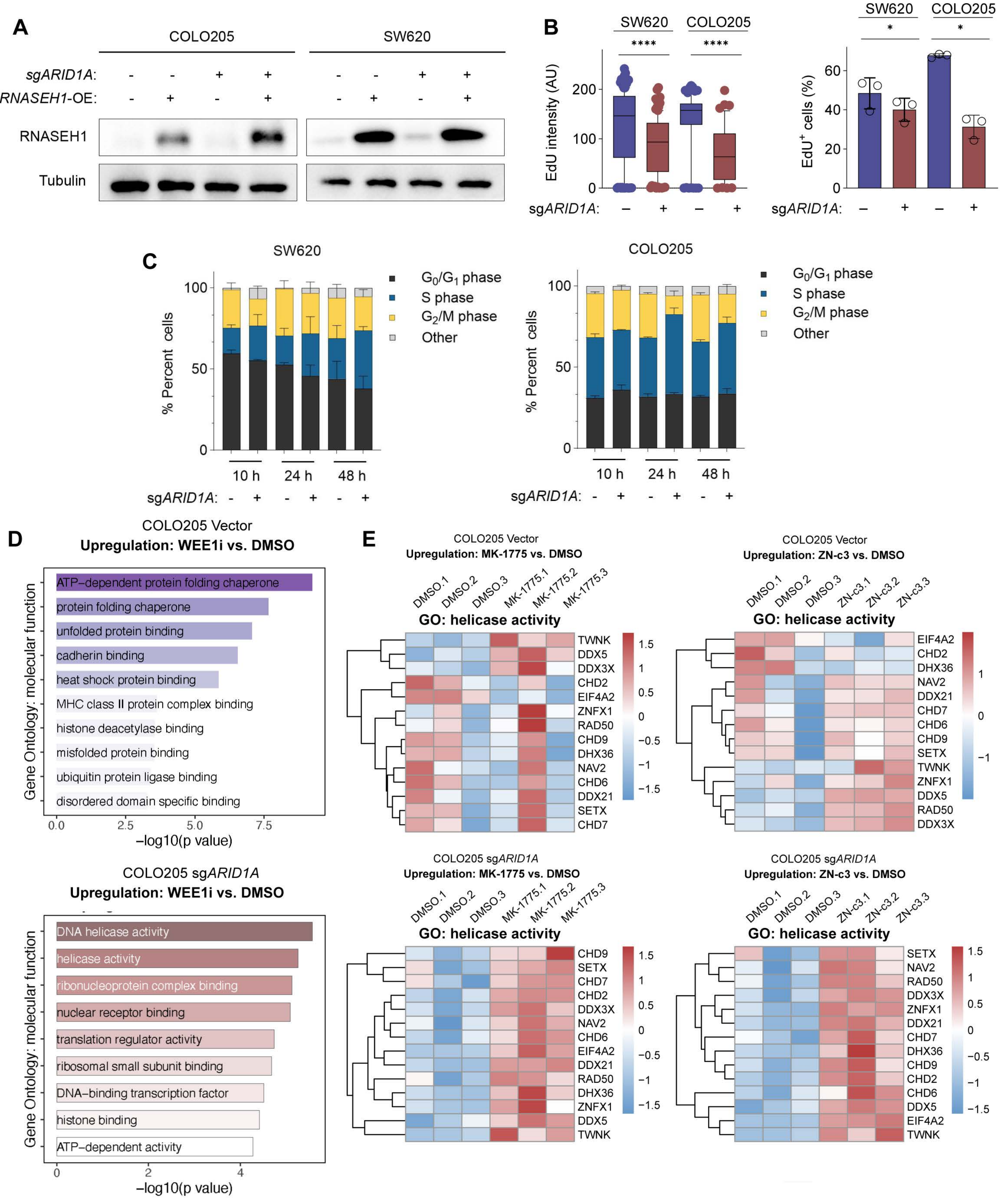

Figure S5. **(A)** Immunoblot analysis confirming RNASEH1 overexpression in COLO205 and SW620 cells (n = 2). **(B)** Representative quantification of EdU staining in SW620 and COLO205 cells. For EdU intensity, the center line indicates a median value; the boxes and whiskers indicate 25th to 75th and 10th to 90th percentiles, respectively. Points below and above the whiskers are drawn as individual points (two-tailed Wilcoxon rank-sum test). For cell percentages, data are shown as mean ± SD (n = 3; one-tailed, paired Student's t-test). **(C)** Cell cycle analysis was performed at the indicated time points using Propidium (PI) staining in SW620 and COLO205 cells. Error bars represent the standard deviation of 3 independent experiments. **(D)** Gene function enrichment analysis annotated with GO in commonly-upregulated genes after 0.5 μM MK-1775 or 0.5 μM ZN-c3 treatment for 48 h, compared with DMSO in COLO205 Vector (top) or sgARID1A (bottom) cell. **(E)** Heatmaps showing gene expressions of helicase activity in different treatment groups, tests were performed in triplicate. Upper left, COLO205 vector cells treated with DMSO or 0.5 MK-1775. Upper right, COLO205 vector cells treated with DMSO or 0.5 ZN-c3. Lower left, COLO205 sgARID1A cells treated with DMSO or 0.5 MK-1775. Lower right, COLO205 sgARID1A cells treated with DMSO or 0.5 ZN-c3.

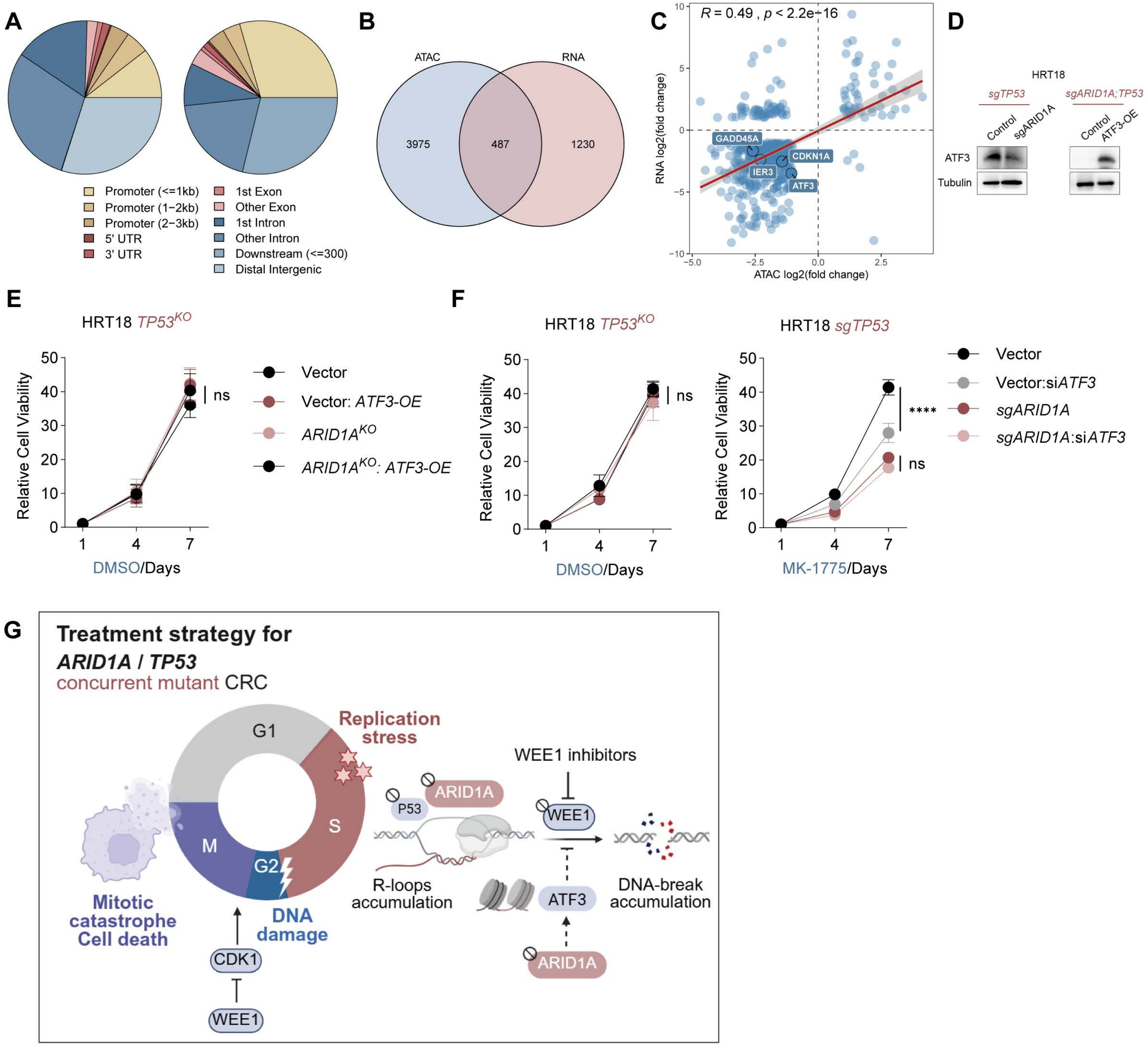

Figure S6. **(A)** Peak annotation in lost and gained accessible peaks in cells above. **(B)** Venn diagram showing overlap differential genes between ATAC-seq and RNA-seq. **(C)** Correlation plots of fold change of overlap differential genes between ATAC-seq and RNA-seq data. **(D)** Immunoblot analysis of ATF3 expression in HRT18 *sgTP53* and HRT18 *sgARID1A;sgTP53* cells (n = 2). **(E)** Cell proliferation assay of HRT18 *sgTP53* cells after ATF-cDNA transfection. **(F)** Cell proliferation assay of HRT18 *sgTP53* cells after siRNA transfection in the indicated days. Left, treated with DMSO; Right, treated with 0.5 μM MK-1775. The results are the representative of three independent experiments, each done in triplicate. Data are shown as mean ± SD. ns, not significant; *****p<0.0001 (two-way ANOVA test). **(G)** A proposed model illustrating the mechanism underlying the sensitivity of *ARID1A/TP53* mutant colorectal cancer cells to WEE1 inhibition. In these concurrent mutant cells, *TP53* loss disrupts the G1/S checkpoint, while WEE1 inhibition compromises the G2/M checkpoint. Simultaneously, *ARID1A* deficiency promotes R-loop accumulation and impairs *ATF3* mediated transcriptional activation of DNA damage repair genes. The convergence of replication stress and checkpoint failure results in excessive DNA damage and ultimately triggers mitotic catastrophe and cell death.

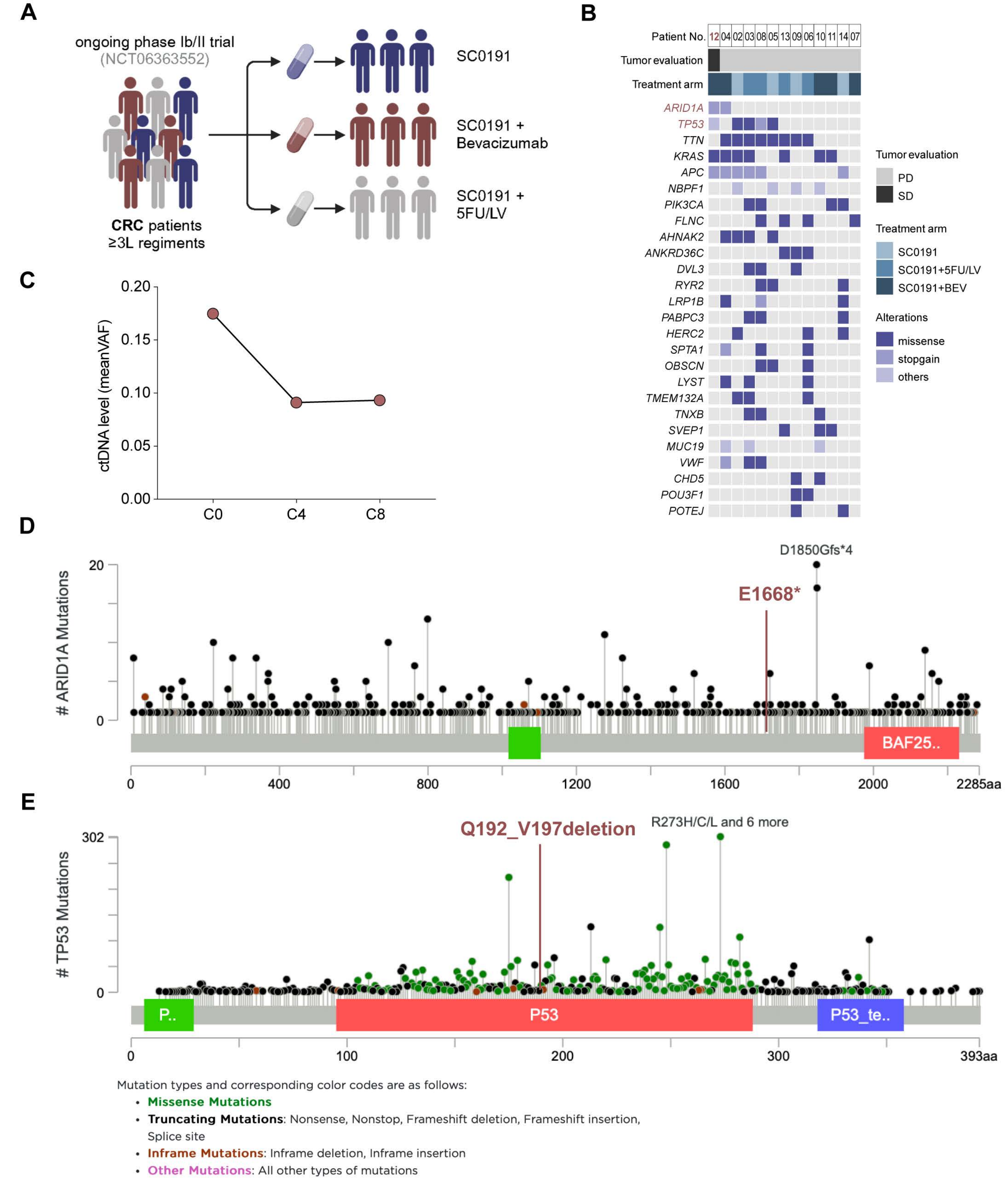

Figure S7. (**A**) Study schedule of SC0191-based clinical trial. (**B**) Mutation profile and clinical efficacy of patients in this trial. (**C**) Dynamic changes of ctDNA levels of patient #12 at Cycle 0 (Baseline), Cycle 4 and Cycle 8. (**D**) *ARID1A* mutation types and corresponding amino acid changes downloaded from OncoKB database. Each vertical bar represents a sample. Location of *ARID1A*^{E1668*} truncating mutation was added in the plot. (**E**) *TP53* mutation types and corresponding amino acid changes. Location of *TP53* Q192_V197 in-frame deletion was added in the plot.

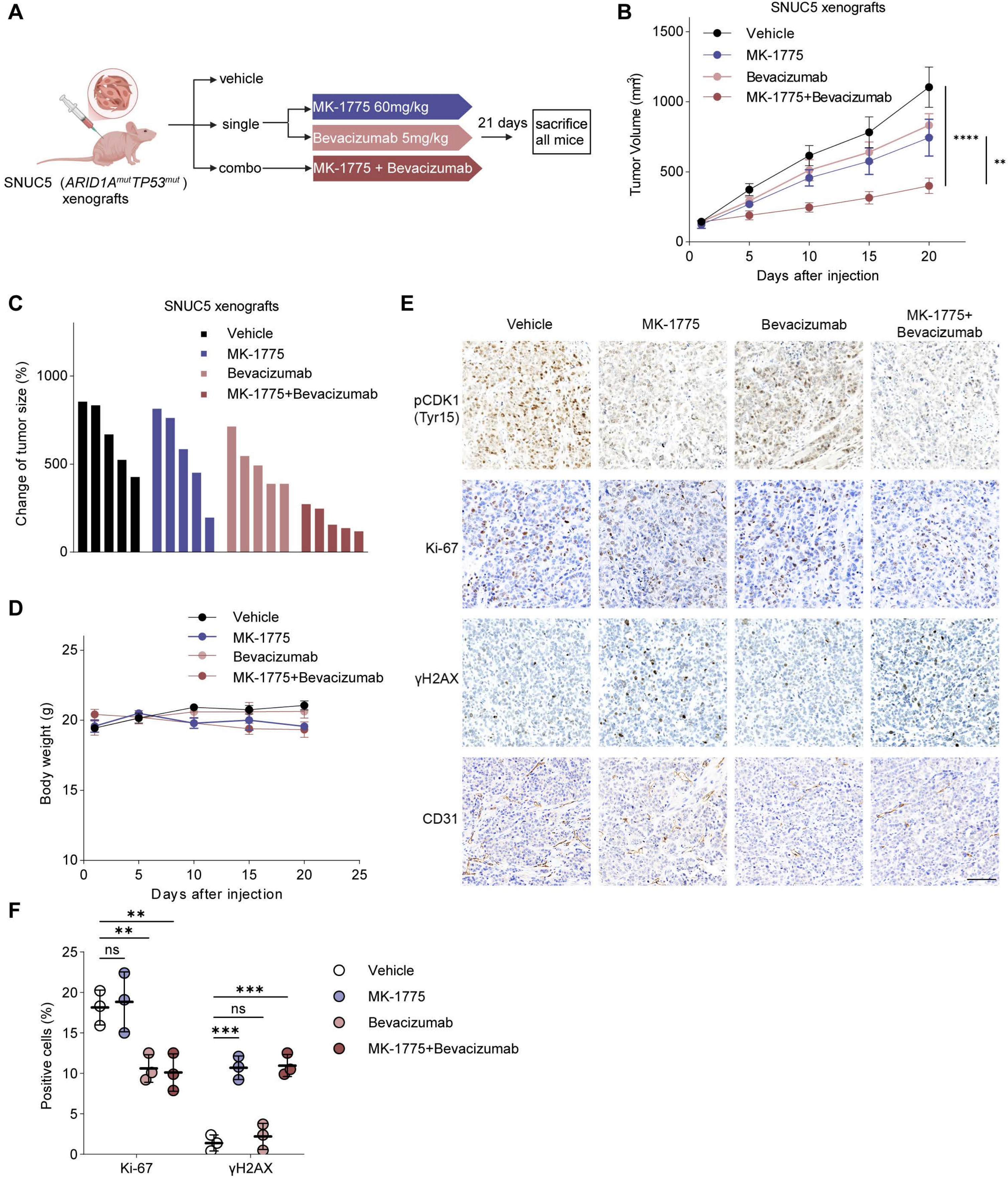

Figure S8. (**A**) Schematic of the in vivo experiment of SNUC5 model. (**B**) Tumor growth curve of SNUC5 xenografts treated with vehicle, MK-1775 (60 mg/kg), bevacizumab (5 mg/kg), or the combination (n = 5). MK-1775 was administered via gavage daily, and bevacizumab was administered by intravenous injection twice a week. Data are shown as mean ± SEM. **p<0.01, ****p<0.0001 (two-way ANOVA test). (**C**) Waterfall plot showing the tumor volume change (at day 20) relative to baseline volume (at day 1). Each bar represents one xenograft tumor. (**D**) Percentage of body weight changes in each group. (**E**) Representative immunohistochemical imagesof pCDK1, Ki-67, γH2AX and CD31 staining of the SUNC5 xenograft tumors. Scale bar = 100 μm. (**F**) Quantification analysis of (**E**). n = 3. Data are shown as mean ± SD. ns, not significant; **p<0.01, ***p<0.001 (two-way ANOVA test).

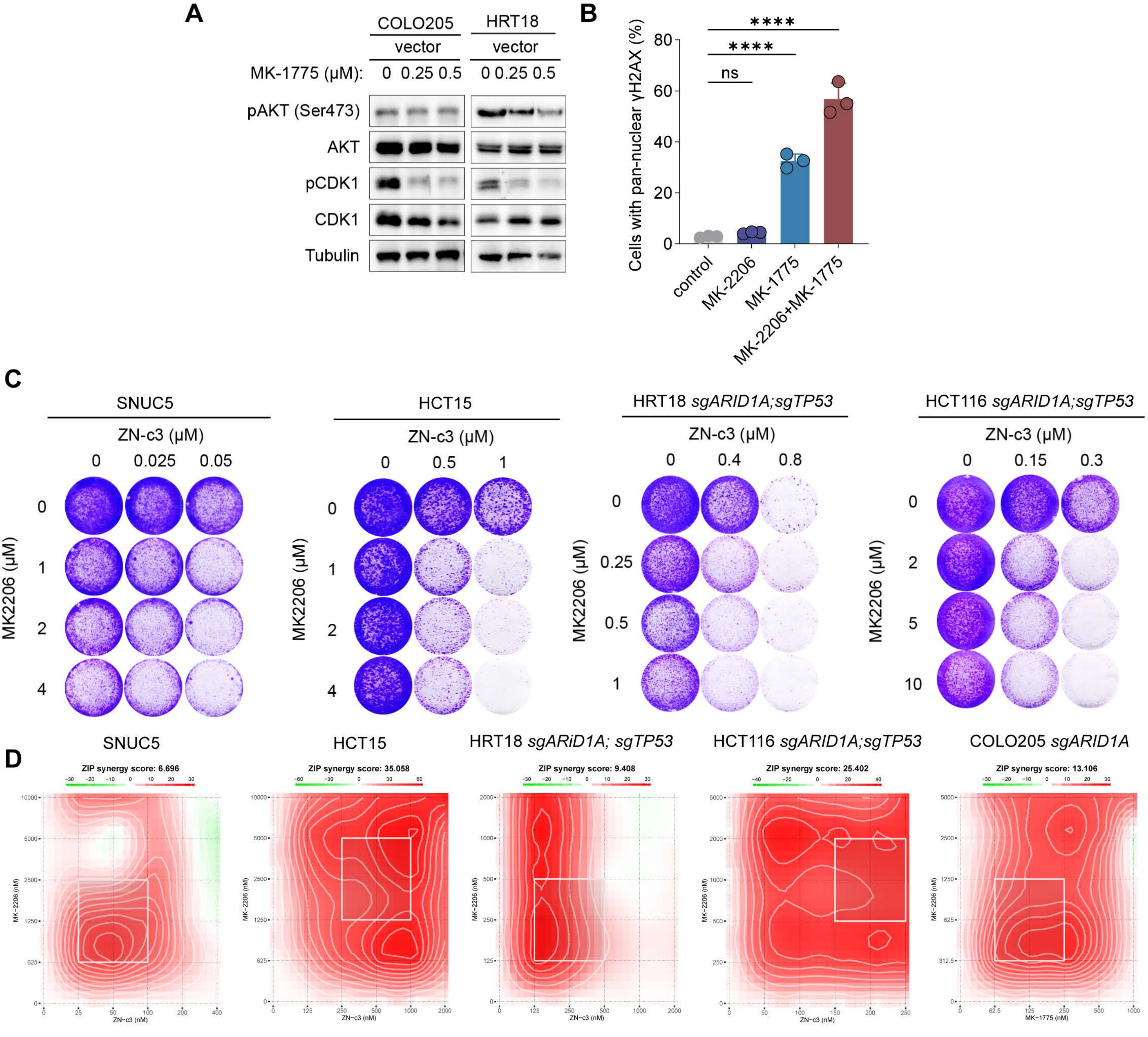

Figure S9. (**A**) Drug response immunoblotting results of COLO205, and HRT18 vector cells treated with 0.25 or 0.5 μM MK-1775 (n = 2).(**B**) Quantification of proportion of pan-nuclear γH2AX cells. data are shwon in mean ± SD. ns, not significant; ****p<0.0001 (one-way ANOVA test). (**C**) Representative results of clonogenic survival assays of HCT15, SNUC5, HRT18 and HCT116 cell lines treated with indicated concentration of ZN-c3 or MK-2206 alone or together for 7-10 days (n = 3). (**D**) Analysis of synergistic effect in ZN-c3 (MK-1775) and MK-2206 combination was performed by SynergyFinder using Zero Interaction Potency (ZIP) model. The inhibition rate was used to calculate ZIP synergy score. The box indicates the most synergistic area.

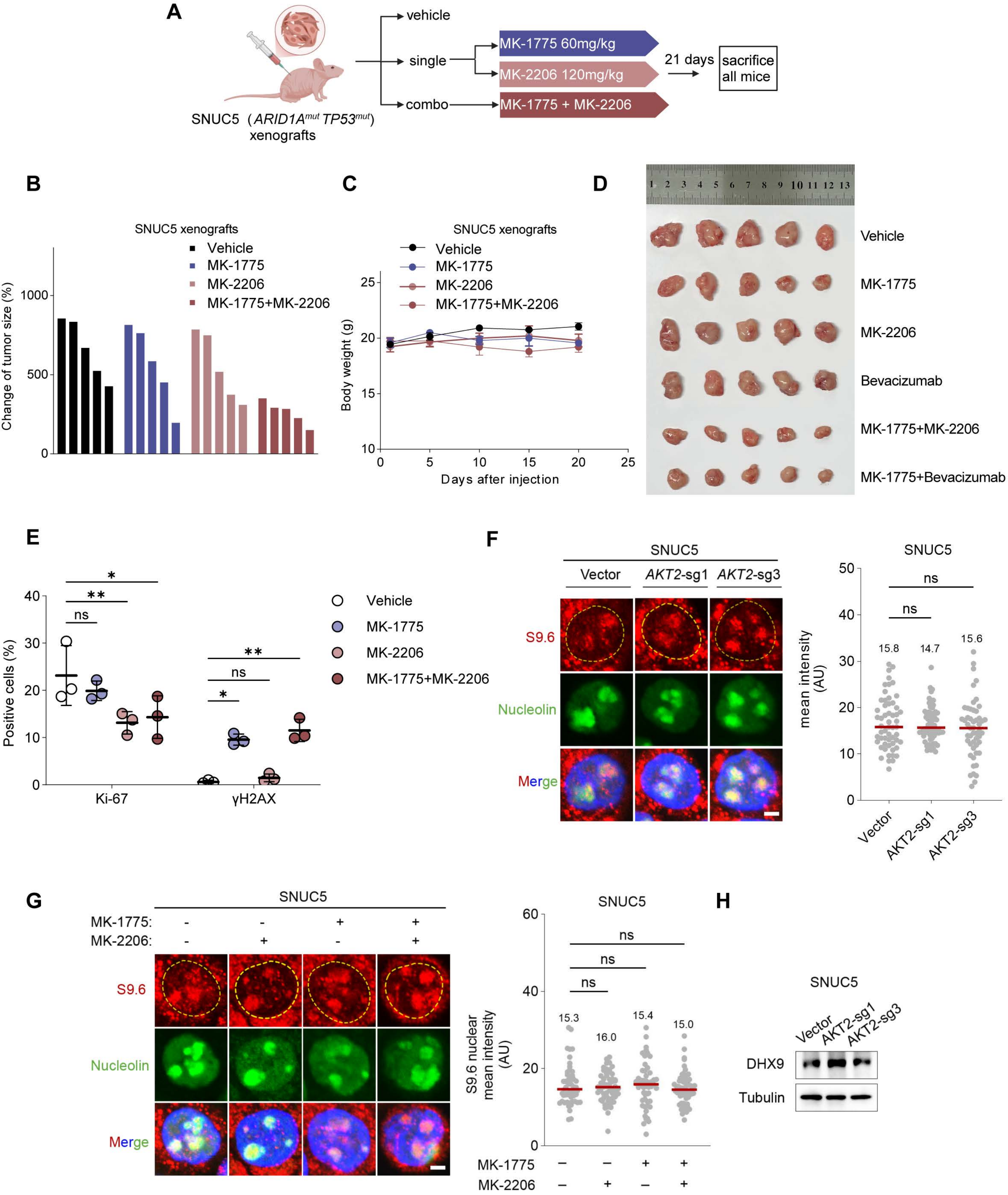

Figure S10. (**A**) Schematic of the in vivo experiment of SNUC5 model. **(B)** Waterfall plot showing the tumor volume change relative to baseline volume. Each bar represents one xenograft tumor. (**C**) Percentage of body weight changes in all four groups of SNUC5 xenografts (**D**) A broad view of SNUC5 xenocraft tumors after euthanizing the mice at endpoint. (**E**) Quantification analysis of IHC results in Figure 7F (n = 3). Data are shown as mean ± SD. ns, not significant; *p<0.05, **p<0.01 (two-way ANOVA test).(**F**) Representative image and quantification of nuclear S9.6 signal intensity in AKT2-KO SNUC5 cells. Scale bar, 5 μm. Data are presented as scatter plots (n = 3). Median values are indicated by red lines. ns, not significant (two-tailed Wilcoxon rank-sum test). AU, arbitrary units. (**G**) Representative image and quantification of nuclear S9.6 signal intensity in SNUC5 cells with the indicated treatment for 24 h. Scale bar, 5 μm. Data are presented as scatter plots (n = 3). Median values are indicated by red lines. ns, not significant (two-tailed Wilcoxon rank-sum test). AU, arbitrary units. (**H**) Immunoblotting results showing DHX9 expression in AKT2-KO SNUC5 cells (n = 2).
